# Probiotic *Bifidobacterium bifidum* strains desialylate MUC13 and increase intestinal epithelial barrier function

**DOI:** 10.1038/s41598-025-92125-2

**Published:** 2025-03-13

**Authors:** Celia Segui-Perez, Liane Z. X. Huang, Fernanda L. Paganelli, Elke Lievens, Karin Strijbis

**Affiliations:** 1 Department of Biomolecular Health Sciences, Division Infectious Diseases and Immunology, Faculty of Veterinary Medicine, Utrecht University, Utrecht, The Netherlands; 2 Winclove Probiotics B.V., Amsterdam, The Netherlands

**Keywords:** Probiotics, *Bifidobacterium bifidum*, *Lactobacillus plantarum*, *Lactiplantibacillus plantarum*, Soluble mucus layer, Intestinal barrier function, Sialic acids, MUC13, Neuraminidase, Tight junctions, Bacteria, Cellular microbiology, Cell polarity, Glycobiology, Gastrointestinal models

## Abstract

Probiotic bacteria including Bifidobacterial species have the capacity to improve intestinal health, but the underlying molecular mechanisms are often not understood. Bifidobacteria are considered keystone species but have a relatively low abundance in the adult intestinal tract. Bifidobacterium colonization depends on degradation of host-derived carbohydrates, including human milk oligosaccharides and mucin-associated oligosaccharides. Specific Bifidobacterium strains can enhance intestinal barrier integrity and improve symptoms of gastrointestinal disorders. We previously reported that the transmembrane mucin MUC13 localizes to the apical and lateral membrane and regulates epithelial tight junction strength. Here, we screened probiotic bacterial strains for their capacity to modulate MUC13 and enhance intestinal barrier function. Of these probiotic bacteria, a *Bifidobacterium bifidum* strain uniquely degraded the MUC13 *O-*glycosylated extracellular domain. Further characterization of two probiotic *B. bifidum* strains (W23 and W28) and the type strain 20456 demonstrated that the W23 and W28 strains adhered strongly to the apical surface, had high sialidase activity, penetrated the mucus layer, and enhanced epithelial barrier integrity. These results underscore the strain-specific properties of these specific *B. bifidum* strains that most likely contribute to their probiotic effects in the intestinal tract.

## Introduction

The intestinal mucus layer acts as a protective barrier limiting bacterial contact with the underlying epithelial cells^1^. In the large intestine, it consists of a “loose” outer layer with which the intestinal microbiota interact and a more compact inner layer that prevents bacterial invasion of the epithelium^2^. In the small intestine, the mucus layer is thinner and consists of a single layer. The primary structural component of the intestinal mucus layer is the gel-forming glycoprotein MUC2. This extensively glycosylated protein forms disulfide-linked dimers, which subsequently undergo polymerization, resulting in the formation of a gel-like network^3^. Additionally, transmembrane mucins including MUC1, MUC3, MUC4, MUC12, MUC13, MUC16, and MUC17 are expressed at the apical surface of the intestinal epithelium^4^. Transmembrane mucins can be barrier proteins that prevent bacterial contact with the epithelium but also play important roles in basic cellular functions such as the regulation of proliferation and cell–cell interactions^4–7^. We previously demonstrated that in addition to its expression on the apical surface, MUC13 also localizes to the upper part of the lateral membrane where the tight junctions are located. Deletion of MUC13 enhanced epithelial barrier strength and reduced the passage of small solutes, while overexpression of MUC13 increased the permeability to ions^8^. In a dextran sodium sulfate (DSS) mouse model, MUC13 expression correlated with increased permeability for 4 kDa FITC-dextran particles^9^. Together, the different intestinal mucins allow bacterial colonization and play an active role in the regulation of epithelial responses to commensal and pathogenic bacteria.

Bifidobacteria are keystone members of the intestinal microbiota. Breast-fed infants develop a bifidobacteria-rich microbiota that includes species such as *Bifidobacterium bifidum, B. breve, B. longum* spp*. infantis,* and *B. longum* spp*. longum*^10,11^. Following the colonization of the infant’s gut, Bifidobacteria contribute to the development of the mucus layer, which is initially underdeveloped^12^. Human milk oligosaccharides (HMOs) are responsible for the colonization and outgrowth of different *Bifidobacterium* strains during infancy^13,14^. Because of the similar structural composition of HMOs and mucin *O*-glycans, HMOs are thought to select for bacteria that can later utilize intestinal mucin *O*-glycans^15^. The intestinal *Bifidobacterium* population is declining upon cessation of breastfeeding but continues to be part of the adult gut microbiome^16^. In the adult intestine, *Bifidobacterium* species support the growth of other commensals^17–19^, modulate T-regulatory responses^20^, improve symptoms of several gastrointestinal conditions^21^ and prevent antibiotic-associated diarrhea^22,23^.

Mucin *O*-linked glycosylation is characterized by the covalent addition of glycans (galactose, *N*-acetylglucosamine (GlcNAc), and *N*-acetylgalactosamine (GalNAc) to the hydroxyl (-OH) group of serine and threonine residues. To cap these structures, sialic acid, fucose, and sulfate are added in the final step^2^. The structure of mucin *O*-glycans is highly heterogeneous, varying across intestinal regions, with the small intestine displaying a less complex *O*-glycosylation pattern compared to the stomach and colon^24,25^. Mucin *O*-glycans shape the composition of the gut microbiota by providing bacterial adhesion sites^26^ and serving as essential nutrient sources. This is especially the case in the distal colon where the availability and accessibility of carbohydrates is limited^27^. The mucin breakdown by gut bacteria is recognized as a normal process of intestinal mucus turnover and starts within the first years of life^28^. *Bifidobacterium* species *B. bifidum, B. longum, B. infantis,* and *B. breve* all have the ability to degrade mucins, while *B. dentium* does not^29,30^. In addition, *B. longum, B. infantis*, and *B. dentium* have been shown to enhance mucin production in both in vitro and in vivo models^31–33^.

Evidence for the beneficial effects of probiotic bacteria on human health is currently on the rise in several research areas. The main characteristics of successful probiotic bacteria in the intestinal tract are their capacity to adhere to the mucus layer and epithelial cells, degrade mucins or enhance mucin production, and increase epithelial barrier functions. While it is known that *Bifidobacterium* species feed on soluble mucins^29,30,34,35^, their interactions with TM mucins have not yet been reported. Because we previously established that MUC13 regulates intestinal barrier functions^8^, we hypothesized that bacterial modulation of MUC13 may exert positive effects on epithelial barrier integrity. Here, we investigated whether probiotic bacteria, including genera that are known to be mucin-degraders, can degrade MUC13 from the surface of the HRT18 intestinal cell line, modulate tight junctions, and affect barrier functions. Our findings demonstrate that two probiotic *Bifidobacterium bifidum* strains W23 and W28 have unique properties compared to the *B. bifidum* type strain. They are highly adherent to the epithelial surface, have high sialidase activity, cleave the MUC13 extracellular domain, and significantly increase epithelial barrier integrity.

## Results

### Strain-specific modification of the extracellular domain of MUC13 by *Bifidobacterium bifidum* in HRT18 cells

**Fig. 1:**
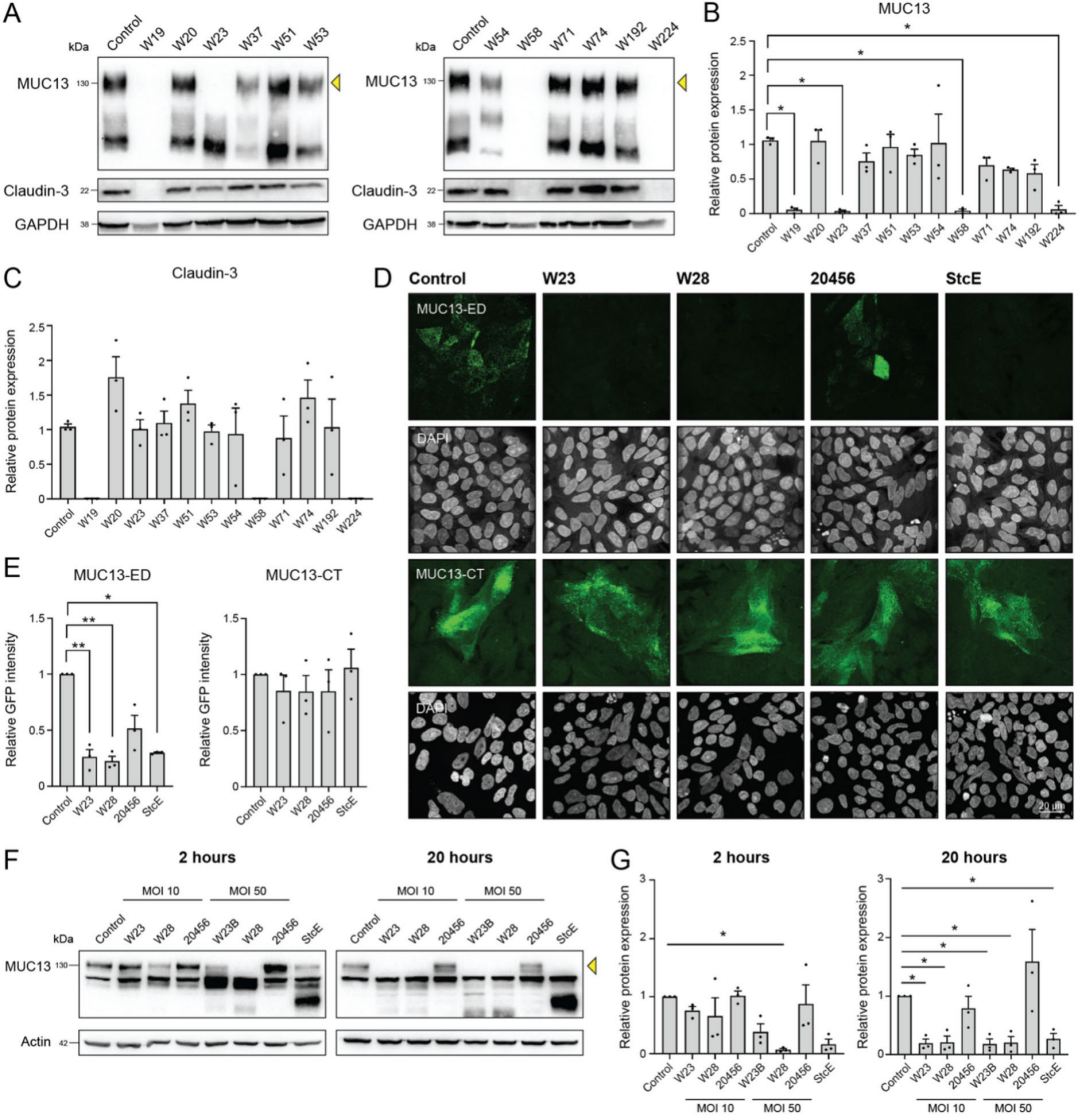
Probiotic *Bifidobacterium bifidum* strains uniquely modify the MUC13 extracellular domain. (**A**) Immunoblot analysis of MUC13 modification/degradation and claudin-3 expression in HRT18 monolayers after incubation with different probiotic strains at MOI 10 for 20 h. The full-length 130 kDa MUC13- band that is used for quantification in (**B**) is marked by an arrow. (**B**,**C**) Quantification of protein expression of the 130 kDa MUC13 and 22 kDa claudin-3 bands (relative to uninfected control) in three biological replicates as depicted in A. All immunoblots used for quantification are available in Figure S1 and Figure S2. Statistical analysis was performed on non-normalized data using a one-way ANOVA with Dunnett’s post hoc test to compare each bacterial sample to the uninfected control. * *p* < 0.05. (**D**) Fluorescence confocal microscopy images of HRT18 cells stained in green with MUC13-ED (above) and MUC13-CT (below) antibodies after 20 h incubation with *B. bifidum* strains at MOI 10 and StcE. The maximal intensity projection is depicted. White scale bars represent 20 µM. (**E**) Quantification of GFP signal in cells stained with MUC13-ED and MUC13-CT antibodies as in (**C**). Statistical analysis was performed on non-normalized data using a one-way ANOVA with Tukey’s post hoc test. * *p* < 0.05; ** *p* < 0.01. (**F**) Immunoblot analysis of degradation of high-MW MUC13 (130 kDa) after 2 and 20 h incubation with *B. bifidum* W23, W28, and 20456 strains at MOI 10 and 50 in HRT18 cells. The full-length 130 kDa MUC13- band that is used for quantification in (**G**) is marked by an arrow. (**G**) Quantification of protein expression of MUC13 (relative to uninfected control) in three biological replicates as depicted in F. All immunoblots used for analysis are available in Figure S3. Statistical analysis was performed on non-normalized data using a one-way ANOVA or Krustal-Wallis test (when the data was not normally distributed) with Dunnett’s post hoc test to compare each bacterial sample to the uninfected control. * *p* < 0.05. Bacteria are listed in Table 1. All graphs depict the SEM of three independent experiments.

MUC13 is a regulator of intestinal tight junction strength^8^. We hypothesized that probiotic bacteria might interact with MUC13 and thereby strengthen epithelial barrier function. 12 bacterial strains belonging to *Lactobacillus*, *Lactiplantibacillus, Limosilactobacillus, Lacticaseibacillus, Lactococcus*, *Bifidobacterium*, and *Enterococcus* genera were selected for their probiotic potential. The bacteria were cultured under anaerobic conditions followed by incubation with confluent monolayers of intestinal HRT18 cells at MOI 10 for 20 h under anaerobic conditions. We then determined MUC13 expression and size by immunoblot, as well as expression levels of the tight junction protein claudin-3, an important barrier-forming protein that seals the paracellular pathway^36^. Incubation of the intestinal monolayer with three different *Lactococcus lactis* strains resulted in the complete removal of both MUC13 and claudin-3 (Fig. 1A-C). After the 20 h incubation with these strains the medium was acidified, a well-known feature of *L. lactis* strains. Therefore, we assumed that the observed effects on MUC13 and claudin-3 were a deleterious effect of the growth conditions. Incubation with *Bifidobacterium bifidum* W23 led to the specific loss of the MUC13 high-molecular weight band (130 kDa) which is the fully *O*-glycosylated MUC13 protein, while a lower molecular weight MUC13 product was still present (Fig. 1A, B). Claudin-3 expression was unaltered by incubation with *Bifidobacterium bifidum* W23 (Fig. 1A, C) and no acidification of the medium was observed. Because of these interesting observations, we decided to select the *B. bifidum* W23 strain for further characterization.

To determine the potential unique properties of *B. bifidum* W23, we selected two additional *B. bifidum* strains, the probiotic W28 and the type strain DSM 20456 (this “type” or reference strain was the first to be sequenced and published) for comparison. First, we studied the capacity of the three *B. bifidum* strains to modulate MUC13 by immunofluorescence. HRT18 intestinal monolayers were incubated with the bacteria and MUC13 was stained using two different antibodies: one directed against the extracellular domain (MUC13-ED) and the other to the cytoplasmic tail (MUC13-CT). We observed that the W23 and W28 strains, unlike the 20456 type strain, effectively reduced MUC13-ED staining, to comparable levels as the *Escherichia coli* O157:H7 StcE protease which is known to cleave *O*-glycosylated domains of mucins (Fig. 1D, E). Moreover, none of the *B. bifidum* strains affected MUC13-CT staining (Fig. 1D, E), suggesting that W23 and W28 have the unique capacity to modulate the extracellular domain of MUC13.

To investigate the dynamics of bacterial modulation of MUC13, we incubated the monolayers with bacteria at different MOIs (10 and 50) and for different durations (2 h and 20 h). Incubation with the W28 strain resulted in MUC13 modulation after 2 h at the lower MOI 10, and at the higher concentration (MOI 50) MUC13 modulation was visible after incubation with both W23 and W28 strains after 2 h (Fig. 1F, G). After 20 h incubation, both W23 and W28 cleaved MUC13 at MOI 10 and 50. Incubation with StcE led to the removal of the 130 kDa MUC13 band and a lower band appeared. In line with the IF experiments, the 20456 type stain did not induce detectable changes in MUC13. These results demonstrate that the modification of the MUC13 extracellular domain occurs fast after incubation with the probiotic *B. bifidum* W23 and W28 strains and perhaps indicates the involvement of enzymes that are already expressed on the bacterial surface.

### *Bifidobacterium bifidum* strains W23 and W28 have high sialidase activity and desialylate MUC13

**Fig. 2:**
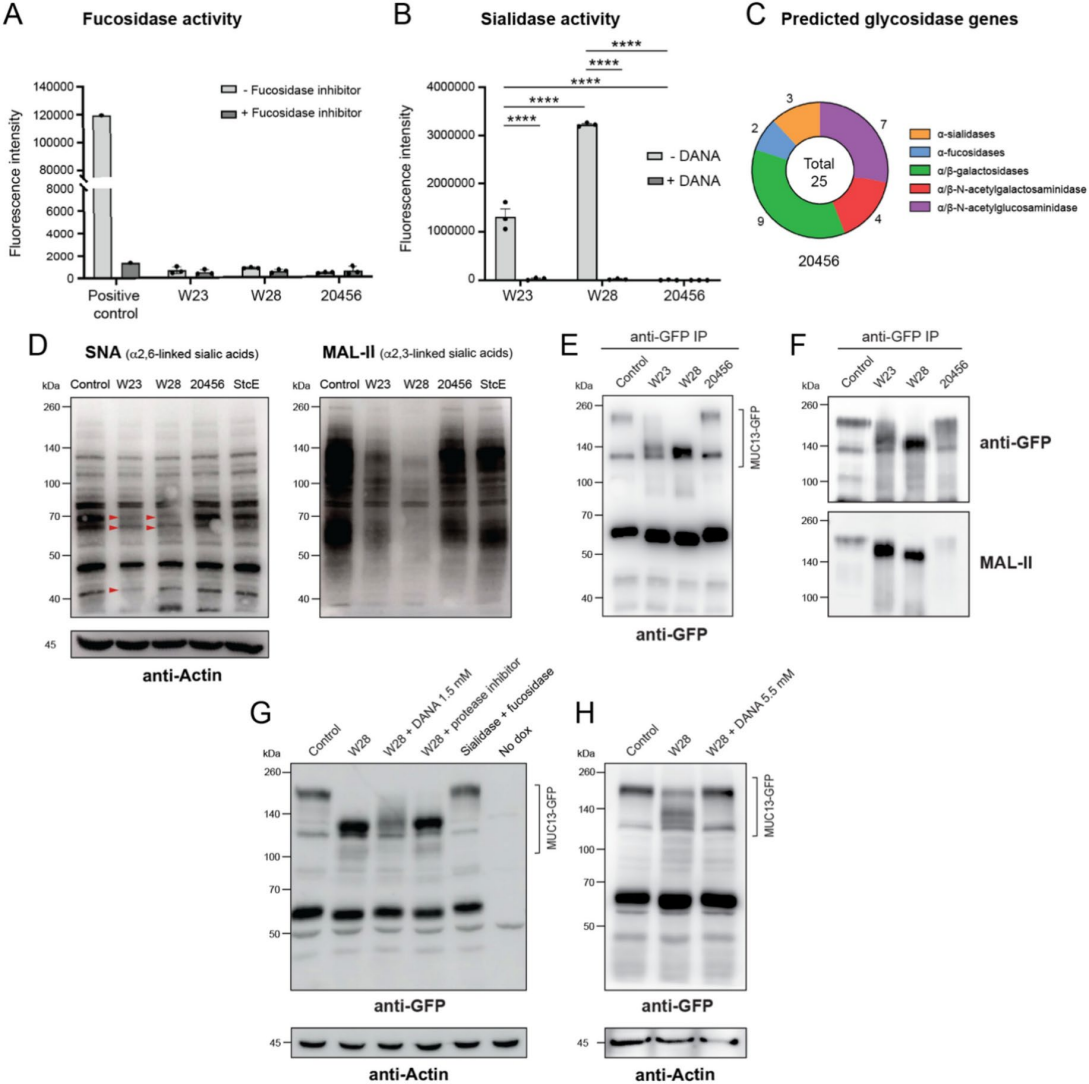
*Bifidobacterium bifidum* strains W23 and W28 have high sialidase activity that results in desialylation of surface proteins including MUC13. (**A**) Fucosidase and (**B**) sialidase activities in bacterial pellets of the *B. bifidum* strains. Graphs depict experimental results of three independent biological replicates. The commercial α1,2,3,4,6-L-fucosidase (Megazyme, E-FUCHS) was used as a positive control for fucosidase activity. All graphs depict the SEM of three independent experiments. Statistical test: two-tailed independent *t*-test. **** *p* < 0.0001. (**C**) Number of identified *O*-glycan-targeting CAZymes in the genome of *B. bifidum* strains 20456. (**D**) Western blot analysis of sialic acid-containing proteins in the HRT18-∆MUC13 + pMUC13 cell line after 2 h incubation with UV-inactivated *B. bifidum* strains at MOI 50 probes with lectins SNA (α-2,6 sialic acids) and MAL-II (α-2,3 sialic acids) lectins. Red arrows mark evidently reduced bands after incubation with W23 and W28 compared to the other lanes. Similar results were obtained in two independent experiments. (**E**) Anti-GFP immunoprecipitation and immunoblot analysis of MUC13-GFP (~ 160 kDa) from HRT18-∆MUC13 + pMUC13 cells line after incubation with UV-inactivated *B. bifidum* at MOI 50 for 2 h. (**F**) Anti-GFP immunoprecipitation and immunoblot analysis of MUC13-GFP with MAL-II lectin after incubation with UV-inactivated *B. bifidum* strains at MOI 200 for 5 h. All immunoblots generated for experiments depicted in (**D**–**F**) analysis are available in Figure S4. (**G**) Immunoblot analysis of MUC13-GFP and actin from HRT18-∆MUC13 + pMUC13 cells incubated with UV-inactivated W28 bacteria at MOI 50 for 4 h. Conditions also included addition of 1.5 mM sialidase inhibitor (DANA) or 1 × Halt protease and phosphatase inhibitor cocktail (p.i.). Cells were also treated with a combination of 200 U/mL of α2,3,6,8,9 neuraminidase A and 0.6 U of α1,2,3,4,6-L-fucosidase. (**H**) Immunoblot analysis of MUC13-GFP and actin from HRT18-∆MUC13 + pMUC13 cells incubated with UV-inactivated W28 bacteria at MOI 120 for 5 h in the absence or presence of 5.5 mM DANA. All immunoblots generated for experiments depicted in (**G**,**H**) are available in Figure S5.

*Bifidobacteria* have a high capacity for degradation of mucin *O*-glycans and we hypothesized that the observed modification of the MUC13 extracellular domain could relate to the activity of glycosidases. To investigate if the three *B. bifidum* strains could indeed perform the initial steps of mucin *O*-glycan degradation, we determined sialidase and fucosidase activities on the bacterial surface after overnight growth. None of the three *B. bifidum* strains exhibited detectable fucosidase activity under the growth conditions tested (Fig. 2A). This was unexpected, as fucosidase activity has previously been reported for *B. bifidum* strains^17,37^. Sialidase activity, on the other hand, was high in strain W23 and highest in strain W28 (Fig. 2B). The sialidase activity of both strains could be inhibited by the sialidase inhibitor DANA (Fig. 2B). Strain 20456 lacked sialidase activity under the growth conditions tested. Because of this surprising lack of activity, we analyzed the genome of *B. bifidum* 20456 strain for the presence of carbohydrate-active enzymes (CAZymes) using the dbCAN2 meta-server pipeline for automated CAZyme annotation^38^. We selected CAZyme genes that were predicted by at least two out of three annotation tools. Detected CAZyme genes included glycoside hydrolases (GH), carbohydrate esterases (CE), glycosyl transferases (GT), carbohydrate-binding modules (CBM), and auxiliary activities (AA). Within the glycoside hydrolase category, the 20456 strain encoded three sialidases, two of which contained a signal peptide, as well as two fucosidases, one of which also contained a signal peptide (Fig. 2C), suggesting that these proteins may be translocated to the bacterial surface or secreted into the environment. However, despite the presence of sialidase and fucosidase genes in the genome of 20456 strain, they were not expressed under the growth conditions tested, highlighting the importance of experimentally testing enzymatic activities.

Next, we determined the capacity of the bacterial sialidases to cleave sialic acids from the epithelial surface. HRT18 monolayers were incubated with UV-killed *B. bifidum* strains at MOI 50 for 2 h followed by analysis of the total pool of sialylated proteins by western blotting. Probing with the lectin SNA, which binds to α-2,6 sialic acids, showed small differences between the strains, as several bands disappeared in the W23 and W28-treated cells (at approximately 70, 65, and 40 kDa) compared to control, 20456, and StcE-treated cells (Fig. 2D). Probing with MAL-II, which detects α-2,3 sialic acids and sulfated glycans^39,40^, revealed a substantial reduction of sialylated proteins in monolayers incubated with the W23 and W28 strains, but not with the 20456 strain (Fig. 2D). These results demonstrate that while the three strains have a similar genetic potential to remove sialic acids, only strains W23 and W28 have constitutive high sialidase activity, which is especially efficient at removing α-2,3 sialic acids from the epithelial surface.

To further investigate the modification of MUC13 by the probiotic *B. bifidum* W23 and W28 strains and the potential role of sialidases, we performed pulldown experiments with a previously established cell line expressing MUC13 tagged with GFP at the C-terminus (HRT18-∆MUC13 + pMUC13)^8^. The cells expressing MUC13-GFP were grown into a monolayer and incubated with UV-killed bacteria for 2 h at MOI 50 followed by pulldown of MUC13-GFP using magnetic beads coated with anti-GFP antibodies.

The full-length unmodified MUC13-GFP protein was detectable as a specific band of about 200 kDa in the control and 20456 elution fractions. As previously observed, both the W23 and W28 strains led to a downward shift of the MUC13-specific band now running around 130 kDa (Fig. 2E). Another GFP-reactive band of about 60 kDa was also detectable, which could be a cleaved or processed form of MUC13. To determine the glycosylation status of the MUC13-GFP products, the elution fraction blot was probed with the MAL-II lectin that recognizes α-2,3-linked sialic acids. The full-length MUC13-GFP and the lower molecular weight products resulting from incubation with W23 and W28 were all recognized by MAL-II, suggesting they all (still) contain α-2,3-linked sialic acids or possibly sialylated glycans that can also be recognized by MAL-II (Fig. 2F). Next, we inhibited bacterial sialidase activity and investigated the modification of MUC13. Incubation of strain W28 with the sialidase inhibitor DANA at 1.5 mM for 4 h prevented the formation of the lower molecular weight MUC13 band and instead led to a smear of multiple MUC13-reactive products. A protease inhibitor did not prevent MUC13 modification by W28. Addition of a mixture of bacterial sialidase and fucosidase enzymes did not modify the high-MW MUC13 band (Fig. 2G). We hypothesized that the observed MUC13 smear in the presence of the inhibitor could point at partial inhibition of the bacterial sialidases. Therefore, we conducted a follow-up experiment with an even higher DANA concentration of 5.5 mM. In this experiment, addition of W28 led to partial modification of the MUC13 band and addition of the DANA inhibitor completely prevented the formation of the lower molecular weight MUC13 band (Fig. 2H). These results demonstrate that sialidase activities of W23 and W28 are important for the observed modification of the MUC13 extracellular domain. However, other glycosidases and/or proteases may also be involved, and the modified MUC13 product is not completely desialylated. W23 and W28 strains differ from the type strain 20456 in their high sialidase activity, which targets a wide range of sialic acid-containing surface proteins, including MUC13.

### Interactions of *Bifidobacterium bifidum* strains with the mucosal surface

**Fig. 3:**
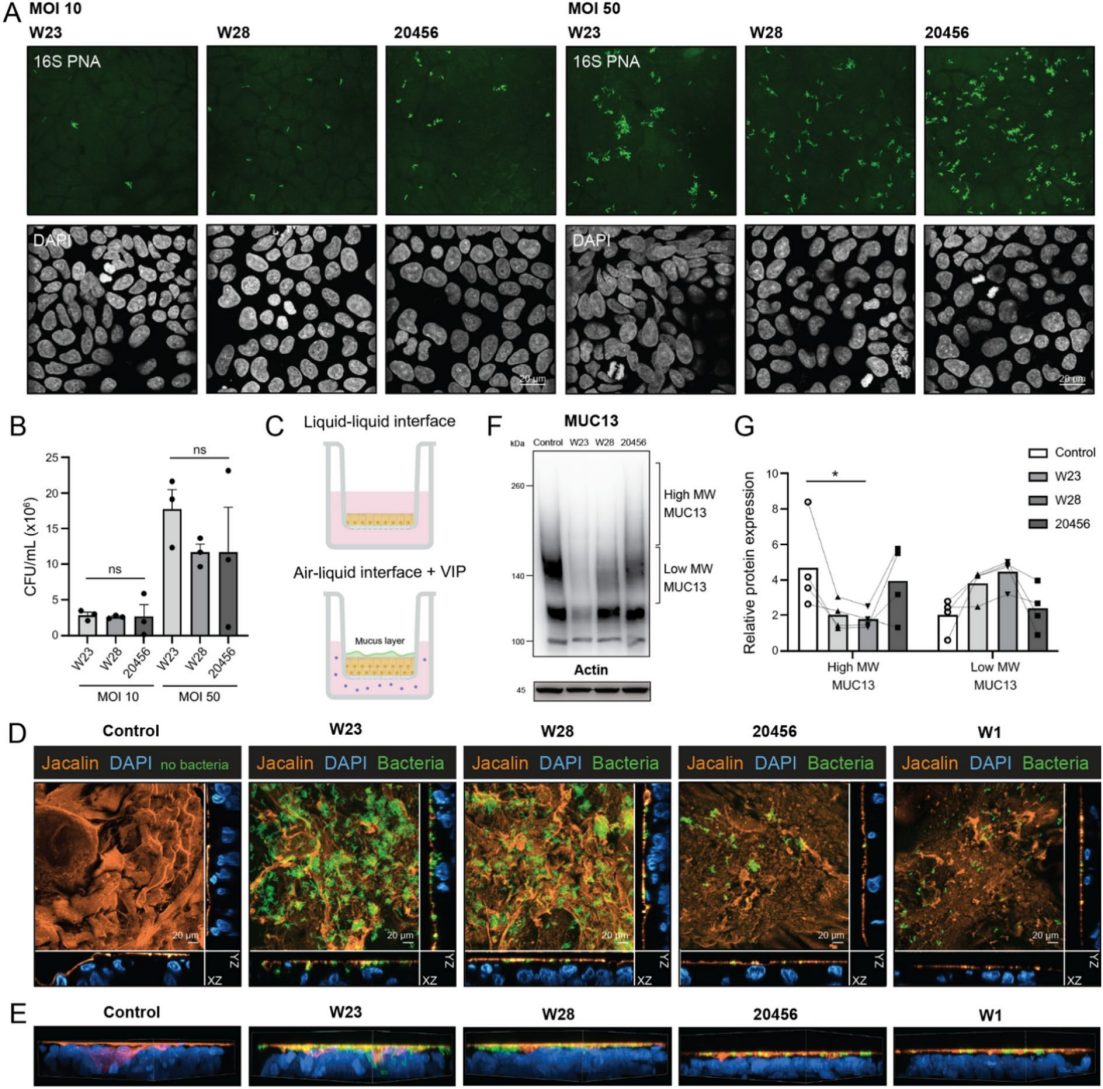
Adherence of *Bifidobacterium bifidum* strains to HRT18 monolayers and penetration of the secreted mucus layer in Caco-2 ALI/VIP cultures. (**A**) FISH staining in combination with confocal microscopy of UV-killed *B. bifidum* adhesion to HRT18 monolayers at MOI 10 and 50 for 20 h using a 16S PNA probe (green). White scale bars represent 20 µM. (**B**) Adhesion of *B. bifidum* W23, W28, and 20456 strains to HRT18 monolayers assessed by quantification of colony-forming units (CFUs). The graph represents the average and SEM of three independent experiments. Statistical test: one-way ANOVA with Tukey’s correction. (**C**) Schematic representation of Caco-2 cells cultured in Transwells under liquid–liquid interface (LLI) and air–liquid interface (ALI) with the basolateral addition of vasointestinal peptide (VIP). Caco-2 ALI/VIP cultures produce a secreted mucus layer on the apical surface^41^ (**D**) Fluorescence confocal microscopy images of Caco-2 cells grown under ALI/VIP conditions to induce mucus formation incubated with pre-stained W23, W28, 20456, and W1 bacteria (green) at MOI 50 for 4 h. The secreted mucus layer was stained with the lectin Jacalin (orange) and DAPI was used for the nuclei (blue). The middle of the image shows the maximal intensity projection of the mucus and bacterial stainings, while the orthogonal view depicts single planes for clarity. White scale bars represent 20 µM. (**E**) 3D rendering of images is shown in (**B**). (**F**) Immunoblot analysis of MUC13 modification in the Caco-2 ALI/VIP cultures after incubation with *B. bifidum* strains at MOI 50 for 4 h. All immunoblots used for quantification are available in Figure S6. (**G**) Quantification of protein abundance of the high and low MW MUC13 (relative to actin) as shown in (**D**). Graph depicts experimental results of three independent biological replicates. Statistical test: one-way ANOVA with Dunnett’s post hoc test. * *p* < 0.05.

Adhesion to the intestinal epithelium and interaction with the secreted mucus layer are important characteristics of probiotic bacteria allowing them access to mucin *O*-glycans. To investigate the capacity of the strains to adhere to the intestinal epithelium, we conducted two types of adhesion assays with HRT18 intestinal monolayers. Because the three strains have different growth speeds and this characteristic would influence the experimental outcome, we first chose to UV-inactivate the bacteria which prevented growth and allowed us to directly access their adhesion potential. UV-inactivated bacteria were incubated overnight (20 h) with the monolayers at MOI 10 and 50, washed, and attached bacteria were visualized using fluorescent in situ hybridization (FISH) using a general 16S peptide nucleotide acid (PNA) probe (EUB338-AF488). Similar amounts of adhered bacteria were observed for all three strains (Fig. 3A). In a second type of adhesion assay, live bacteria were incubated with HRT18 monolayers for 2 h, followed by washing steps, and quantification of adherent bacteria by plating and quantification of colony-forming units (CFUs). In line with the FISH stainings, no significant differences in CFUs were observed between the bacteria at both MOI 10 and MOI 50 (Fig. 3B). We conclude that the three *B. bifidum* strains have a similar capacity to adhere to an intestinal epithelial surface. In vivo, intestinal bacteria interact with the secreted mucus layer that mostly consists of the soluble mucin MUC2. We recently developed a novel in vitro Caco-2-based model with a secreted MUC2 mucus layer^41^. In this model, Caco-2 cells are seeded on Transwell plates, the medium in the inner compartment is removed to induce air–liquid interface (ALI) conditions, and vasoactive intestinal peptide (VIP) is added to the basolateral compartment (Fig. 3C). Culturing Caco-2 cells under ALI/VIP conditions induces expression and secretion of soluble mucin MUC2 and transmembrane including MUC13 are highly expressed. Therefore, we selected this model to study how our *B. bifidum* strains interact with the secreted mucus layer and MUC13.

Caco-2 ALI/VIP cultures were incubated with bacteria that were pre-stained with the fluorescent dye carboxyfluorescein succinimidyl ester (CFSE) and the mucus layer was stained with the glycoprotein-binding lectin Jacalin. As a control, we included the *Lactiplantibacillus plantarum* W1 strain (also known as WCFS1, formerly *Lactobacillus plantarum*), which has been previously shown to colonize the mucus layer in this Caco-2 model^41^. Incubation of the Caco-2 ALI/VIP cultures with bacteria at MOI 50 for 4 h demonstrated that strains W23 and W28 highly adhered to the Jacalin-positive secreted mucus layer and that strains 20456 and W1 colonized the mucus layer to a much lesser extent (Fig. 3D). We also observed that the intensity of the Jacalin staining was reduced in all tissues incubated with bacteria compared to the condition without bacteria. Orthogonal views from Z-stacks indicated that strains W23 and W28 penetrated the mucus layer, reaching deeper and in closer proximity to the epithelial cells compared to strains 20456 and W1 (Fig. 3D, E). Immunoblot analysis of MUC13 showed that incubation with strains W23 and W28 resulted in a modification of MUC13 leading to a reduction in molecular weight as was also observed in the HRT18 experiments (Fig. 3F). The MUC13 pattern in these Caco-2 cultures was more complex compared to the HRT18 monolayers. Therefore, we quantified the bacterial effects by separating the MUC13 band in two sections: one at the higher molecular weight, representing the full *O*-glycosylated MUC13, and the other at a lower molecular weight, indicating deglycosylation or degradation. W23 and W28 induced a similar reduction in intensity of the high MW MUC13 band, but only the W28 data reached significance (Fig. 3G). Together, these results demonstrate that the three *B. bifidum* strains adhere to epithelial surface to a similar extend, but that strains W23 and W28 are more capable of colonizing the secreted mucus layer and can also modulate the TM mucin MUC13 at the apical surface of the epithelium.

### Increased barrier integrity by *Bifidobacterium bifidum* strains W23 and W28

**Fig. 4:**
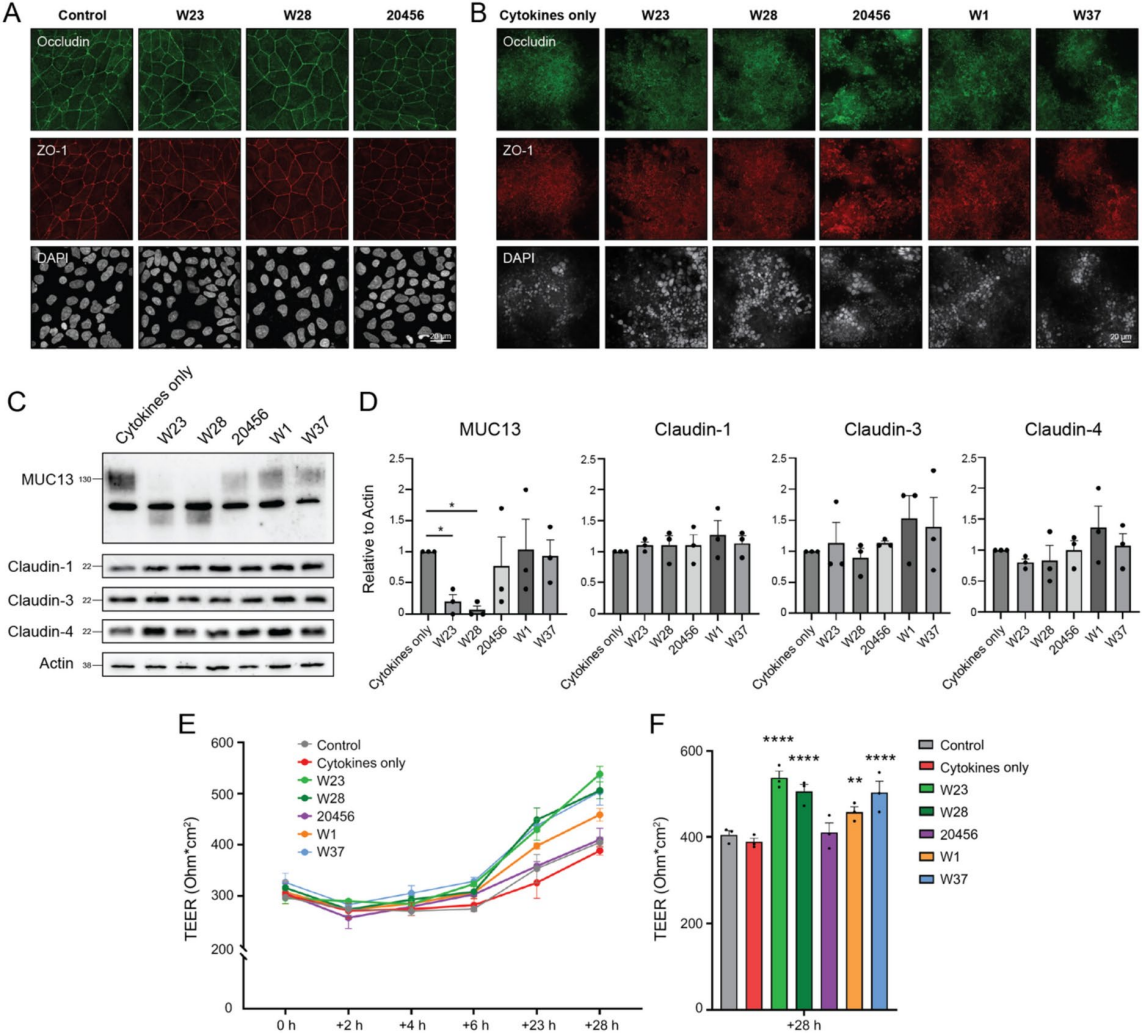
*Bifidobacterium bifidum* strains W23 and W28 enhance intestinal barrier properties. (**A**) Immunofluorescence confocal images of HRT18 cells grown in glass slides until full confluency and infected with *B. bifidum* strains W23, W28, and 20456 at MOI 10 for 20 h. Cells were stained for occludin (green), ZO-1 (red), and DAPI (white). The maximum intensity projection is depicted. White scale bars represent 20 µM. (**B**) Immunofluorescence images of HRT18 cultures grown in Transwells incubated with W23, W28, 20456, W1, and W37 at MOI 100 for 28 h. Cultures were stained for occludin (green), ZO-1 (red), and DAPI (white). Z-stacks were captured from the apical surface until the appearance of nuclei and maximum intensity projections are depicted. White scale bars represent 20 µM. (**C**) Immunoblot analysis of MUC13 modification and expression of claudins-1, -3, and -4 in HRT18 Transwell cultures after incubation with bacterial strains W23, W28, 20456, W1, and W37 at MOI 100 for 28 h and during a challenge with 100 ng/mL IL-1β and 100 ng/mL TNF-α. The control sample only received the cytokine challenge without bacteria. All immunoblots used for quantification are available in Figure S7. (**D**) Quantification of protein expression of MUC13 and claudins-1, -3, and -4 (relative to actin) as shown in (**C**). All graphs depict the SEM of three independent experiments. Statistical analysis was performed on non-normalized data using a one-way ANOVA with Dunnett’s post hoc test to compare each bacterial sample to the uninfected control. * *p* < 0.05. (**E**) TEER measurements of HRT18 Transwell cultures over time during incubation with W23, W28, 20456, W1, and W37 strains at MOI 100 and cytokine challenge at baseline as described for C. (**F**) TEER values at the 28 h timepoint depicted in E. Graphs depict SEM of three independent biological replicates. Statistical test: two-way ANOVA with Dunnett’s post hoc test. **** *p* < 0.0001.

To address the possible impact of the different *B. bifidum* strains on intestinal epithelial barrier functions, we first investigated the expression of tight junction proteins. Fully confluent HTR18 cells were incubated with the three *B. bifidum* strains at MOI 10 for 20 h. Incubation with the bacteria did not induce gross changes in occludin and ZO-1 expression or localization (Fig. 4A). In a next set of experiments, we investigated the potentially restorative effects of different bacterial strains on epithelial barrier under a pro-inflammatory stress condition. We made use of a previously established experimental setup with relatively high bacterial MOIs to detect effects on epithelial barrier properties^42,43^. HRT18 monolayers were grown in Transwell plates for 14 days followed by incubation with the bacteria at MOI 100 for 28 h under anaerobic conditions. After the addition of the bacteria, the pro-inflammatory cytokines IL-1β and TNF-α were added apically for 2 h. Additionally, two other probiotic strains were included, the *Lactiplantibacillus plantarum* strain W1/WCFS1 and the *Lactobacillus acidophilus* strains W37). The W1 strain has been previously shown to enhance epithelial barrier strength^42,43^.

First, we assessed cell junction composition in the 14-day HRT18 Transwell cultures incubated with the bacteria. Because these cultures were multilayered with TJs residing at varying depths, we captured Z-stacks and analyzed maximum intensity projections. Incubation for 28 h with the *B*. *bifidum* strains W23, W28, 20456, W1, or W37 at MOI 100 did not lead to significant visual alterations in occludin or ZO-1 expression levels (Fig. 4B). Claudins are essential proteins that are part of the tight junction complex and regulate the paracellular passage of small solutes. We next investigated the impact of our *B. bifidum* strains on claudin expression in Transwell cultures. Incubation with the bacteria did not lead to significant changes in expression of claudin-1, claudin-3, or claudin-4 (Fig. 4C, D). In line with our previous experiments, MUC13 was again modified by W23 and W28, while the *B. bifidum* type strain nor the *Lactiplantibacillus* or the *Lactobacillus* strains significantly affected MUC13.

The total levels of tight junction proteins measured were comparable, but overall tight junction strength can also be determined by the interplay of proteins within the TJ complex. Therefore, we determined whether the probiotic bacteria influenced transepithelial electrical resistance (TEER), which measures permeability to ions and is an important assay to determine overall epithelial barrier function. TEER was measured at different time points during the course of the experiment. In these cultures, addition of IL-1β and TNF-α did not negatively impact TEER compared to the control without cytokines. After 28 h, incubation with the probiotic strains W23, W28, W1, and W37 significantly increased the TEER, while the type strain 20456 did not have a significant effect on TEER (Fig. 4E, F). The restorative effects of strains W23 and W28 were more pronounced than the effect of W1, and comparable to strain W37. We conclude that the probiotic *B. bifidum* W23 and W28 strains can significantly enhance barrier function in this intestinal model system. The overall conclusion of this work is that the probiotic *B. bifidum* W23 and W28 strains have unique properties including strong interaction with the mucosal surface, high sialidase activity, modification of MUC13, and enhancement of intestinal epithelial barrier function that sets them apart from the *B. bifidum* type strain.

## Discussion

In recent years, there has been a growing interest in probiotics and their beneficial effects on human health, but their interactions with the intestinal mucus layer are often not well understood. We have previously reported that the transmembrane mucin MUC13 which is highly expressed on the apical surface of the intestinal epithelium regulates tight junction strength^8^. In this study, we aimed to identify probiotic bacteria that modified MUC13 and could strengthen epithelial barrier function. We uncovered that the two probiotic *B. bifidum* strains W23 and W28 can modify the MUC13 extracellular domain but leave the cytoplasmic tail intact, while the *B. bifidum* type strain DSM 20456 leaves MUC13 unaffected (Fig. 1). The probiotic strains have high sialidase activity that is essential for MUC13 modification, but the shorter MUC13 products are not completely devoid of sialic acids (Fig. 2). They are highly adherent to the intestinal epithelial surface and can efficiently colonize the secreted mucus layer and modify the transmembrane mucins at the epithelial apical surface (Fig. 3). These effects are not harmful to the intestinal epithelium, and incubation with the W23 and W28 strains positively impacts intestinal epithelial barrier integrity under pro-inflammatory conditions (Fig. 4). Based on our previous work with MUC13 knockout cells^8^, we expected that modulation of MUC13 would induce changes in claudin expression. However, in the current experimental setup, there was no significant change in the claudin profile, while other studies did find that *B. bifidum* induced altered expression of junction proteins^44–46^. It could be the case that bacterial modulation of MUC13 does not result in the same effect on tight junction complexes, that the time frame of the current experiments is not sufficient, or that the beneficial effect of the bacteria on the barrier function is caused by different pathways that do not involve MUC13. Because we are limited by the sensitivity of intestinal epithelial cells to anaerobic conditions, more complex model systems are required to follow the effect over a longer period of time. With respect to characterization of bacterial phenotypes, this work underscores the importance of functional assays to determine unique strain-associated characteristics as has been pointed out previously^47–49^.

*B. bifidum* is well-documented for its ability to degrade host-derived *O*-glycans, including HMOs and mucins, through numerous extracellular glycosidases^10,29,50^. Multiple studies have demonstrated the ability of *B. bifidum, B. longum,* and *B. breve* to degrade mucins in vitro^29,30^, but *B. bifidum* was best capable of using mucins or HMOs for growth^10,17,51^. The two probiotic *B. bifidum* strains W23 and W28 in our study exhibited high sialidase activity that acted on multiple sialic acid-containing proteins on the epithelial surface including MUC13 (Fig. 2B, D). Surprisingly, none of the *B*. *bifidum* strains in this study had detectable fucosidase activity in our fluorometric assay (Fig. 2A). The presence of fucosidase activity in *B*. *bifidum* has been shown in literature by measuring the release of fucose from mucins^17^ and by the activity determination of a recombinantly expressed *B*. *bifidum* AfcA fucosidase^37^. This difference might be explained by different substrate specificities for the fluorescent substrate versus mucin-bound fucose moieties. Another explanation could be the difference in culture conditions as the addition of mucins or fucose to the medium might induce fucosidase expression.

The degradation of mucin *O*-glycans by a select group of extracellular glycosidase-producing bacteria facilitates nutritional support for other enteric bacteria, a phenomenon known as cross-feeding. *B. bifidum* liberates mono- and di-saccharides from host glycans and releases them into the environment, where they can be consumed by *B. bifidum* itself or utilized by other bacteria^51,52^. For example, such a mutualistic interaction has been observed for sialic acid consumption between *B. bifidum* and *B. breve*^18^, and for fucose between *B. bifidum* and *Eubacterium halii*^17^. Similar trophic interactions appear between *B. longum* and *B. adolescentis*, which engage in the degradation of plant-derived polysaccharides^19^. These observations point towards a specialization of carbohydrate preference among the different Bifidobacterial species and a fundamental role for *B. bifidum* in sustaining the growth of other *Bifidobacterium* species. Here, we demonstrated that *B*. *bifidum* probiotic W23 and W28 strains are highly adherent and de-glycosylate the intestinal glycocalyx including the human transmembrane mucin MUC13. We hypothesize that *B. bifidum* can utilize these epithelial glycans as a nutrient source, which potentially confers a niche advantage when in close contact with the epithelial cell layer.

Balancing mucin degradation by bacteria with mucin production in the intestine is crucial for maintaining gut health. This soluble mucus layer is constantly being renewed by goblet cells, which secrete mucins in response to various stimuli, including bacteria^53^. *Akkermansia muciniphila* is another well-known mucin-degrading bacterium associated with beneficial effects in humans^54,55^, although other studies have also reported negative impacts on intestinal health under specific conditions^56^. In healthy individuals, there is a dynamic equilibrium between mucus degradation and production. In patients with inflammatory bowel disease (IBD), this balance is often disrupted. Chronic inflammation can lead to mucosal damage and an altered mucus layer^57^ and excessive mucin degradation by bacteria can be detrimental in such conditions^58^. *B. bifidum* can efficiently degrade mucins^51,52^, but at the same time, *B. bifidum* can induce the number of mucin-positive goblet cells and mucin gene expression^59–61^. Moreover, *B. bifidum* showed beneficial effects towards preventing excessive mucus degradation by *A. muciniphila* following small intestine injury in a mouse model^62^, suggesting that the mucus-degradation potential of *B*. *bifidum* can be protective. Indeed, despite the high mucin degradation capabilities of *B. bifidum* W23 and W28 strains, incubation of HRT18 cells with these strains led to increased epithelial barrier function (Fig. 4E, F). This is in line with other studies showing positive effects of Bifidobacterial species on the intestinal epithelial tight junction barrier^44,63^. Therefore, we think that mucus degradation and colonization are part of the beneficial *Bifidobacterium* probiotic-host interactions rather than an undesirable phenotype. Further research is needed using more complex coculture or in vivo experiments to understand the complex interplay between different bacteria and the mucus layer leading to the potential beneficial effects of *B. bifidum* on mucosal health in the presence of the microbiota.

The capacity of probiotic bacteria to adhere to mucus and/or human epithelial cells is an important criterion for in vitro screening^64^. Bacterial adhesion prevents rapid peristaltic elimination^64^, facilitates immune modulation^65^, and can contribute to colonization resistance against invading pathogens^66^. *B. bifidum* adherence to intestinal cells can be mediated by various factors, including the outer surface lipoprotein BopA^67^, sortase-dependent pili^68^, and extracellular glycoside hydrolases^69^. Moreover, *B. bifidum* exhibits a greater number of CBMs compared to other *Bifidobacterium* species^70^, which facilitate *B. bifidum* adherence to cell epithelial glycans. In our study, the three *B. bifidum* strains had equal binding capacity to the epithelial surface when UV-killed (Fig. 3A). However, in more complex interaction experiments with live bacteria and a model including a secreted mucus layer, the probiotic W23 and W28 strains had a greatly increased colonization of the mucosal surface compared to the 20456 type strain (Fig. 3D, E). Bacterial attachment to the mucus layer and their high mucin-degradation potential may facilitate nutrient acquisition and bacterial growth at the mucus cell surface, thereby increasing their potential to exert beneficial effects on the host.

In vitro epithelial models are useful for studying host-microbe interactions and epithelial barrier function. However, many traditional models lack a soluble mucus layer, which is a critical component for achieving a greater resemblance to the intestinal epithelium in vivo. Incorporation of a soluble mucus layer within in vitro models, as we are presenting here, can provide a more physiologically relevant context for the assessment of their potential health effects and mechanism of action. These types of in vitro models allow for the selection of the most promising probiotic strains before moving towards in vivo applications.

## Materials and methods

### Cell lines and culturing

The human intestinal epithelial cell line HRT18 (ATCC-CCL-244) was routinely grown in 25 cm^2^ flasks in Dulbecco’s modified Eagle’s medium (DMEM) + glutamax (Gibco, 31966047) containing 10% fetal calf serum (FCS) (Sigma, F7524) at 37 °C in 10% CO_2_. The Caco-2 cell line (ATCC-HTB-37) was grown in DMEM low glucose (Gibco, 11885084) + glutamax containing 10% FCS at 37 °C in 10% CO_2._ For overexpression of MUC13 tagged with GFP in C-terminal in HRT18-∆MUC13 cells, we used a previously made cell line, HRT18-∆MUC13 + pMUC13. Cells were detached with 0.25% trypsin (ThermoFisher, 25200-072), passaged twice a week in a 1:20 dilution for HRT18, 1:12 for HRT18-∆MUC13 + pMUC13, and 1:14 for Caco-2 cells. Cells were split before they reached 80% confluency. All cell lines were routinely tested for *Mycoplasma* contamination.

### Bacterial strains and culture conditions

The bacterial strains used in this study are listed in Table 1. All bacteria were grown at 37 °C under anaerobic conditions (5% H_2_, 10% CO_2,_ 85% N_2_) in a Coy Lab’s Vinyl Anaerobic Chamber. Bifidobacteria were grown in Bifidobacterial medium following the DSMZ #58 protocol (https://bacmedia.dsmz.de/medium/58?bacdive=1717). The remaining bacteria were grown in De Man, Rogosa, and Sharpe (MRS) medium (Millipore, 69966). To inactivate bacteria, an overnight culture was centrifuged, and the pellet was washed twice with DPBS. The pellet was resuspended in DPBS, set to OD = 1, and divided into a 6-well plate (1 mL/well). The plates were exposed without lid three times to 100,000 µJ/cm^2^ in a UV-crosslinker (Stratagene, Stratalinker 1800 UV) and stored at − 20 °C until use.

**Table 1:** Overview of bacterial strains used in this study.

Species	Former name	Strain	Medium	Reference
*Lactiplantibacillus plantarum*	*Lactobacillus plantarum*	W1/ WCFS1	MRS	Winclove B.V
*Lactoccocus lactis subspecies lactis*		W19	MRS	Winclove B.V
*Lacticaseibacillus paracasei*	*Lactobacillus paracasei*	W20	MRS	Winclove B.V
*Bifidobacterium bifidum*		W23	DSMZ #58	Winclove B.V
*Bifidobacterium bifidum*		W28	DSMZ #58	Winclove B.V
*Lactobacillus acidophilus*		W37	MRS	Winclove B.V
*Bifidobacterium animalis subspecies lactis*		W51	MRS	Winclove B.V
*Bifidobacterium animalis subspecies lactis*		W53	MRS	Winclove B.V
*Enterococcus faecium*		W54	MRS	Winclove B.V
*Lactococcus lactis subspecies lactis*		W58	MRS	Winclove B.V
*Lacticaseibacillus rhamnosus*	*Lactobacillus rhamnosus*	W71	MRS	Winclove B.V
*Lactobacillus helveticus*		W74	MRS	Winclove B.V
*Limosilactobacillus reuteri*	*Lactobacillus reuteri*	W192	MRS	Winclove B.V
*Lactoccocus lactis subspecies cremoris*		W224	MRS	Winclove B.V
*Bifidobacterium bifidum*		DSM 20,456	DSMZ #58	DMSZ

### Antibodies and reagents

For Western blotting and immunofluorescence, antibodies against claudin-1 (ThermoFisher, 51-9000), claudin-3 (ThermoFisher, 34-1700), claudin-4 (ThermoFisher, 32-9400), occludin (Invitrogen, 33-1500), ZO-1 (Abcam, ab216880), MUC13-ED (Abcam, ab235450), MUC13-CT hybridoma supernatant (in house), Jacalin (Vector Laboratories, B-1155-5), anti-GFP antibody (Sigma, SAB4301138), Sambucus Nigra Lectin (SNA, Vector Laboratories, B-1305-2), Maackia Amurensis Lectin II (MAL-II, Vector Laboratories, B-1265-1), GAPDH (Tebu-Bio, ABL1021), and β-actin (Bioss, bs-0061R) were used. Secondary antibodies used for immunoblotting were goat anti-mouse-HRP (Sigma, A2304), goat anti-rabbit-HRP (Sigma, A4914), and streptavidin-HRP (Jackson ImmunoResearch, 016–030-084). Secondary antibodies for immunofluorescence were goat anti-mouse-Alexa488 (ThermoFisher, A11029), goat anti-mouse-Alexa568 (ThermoFisher, A11031), goat anti-rabbit-Alexa488 (ThermoFisher, A11034), goat anti-rabbit-Alexa568 (ThermoFisher, A11036), and DAPI (D21490, Invitrogen). For bacterial staining, the fluorescein-based dye CFSE (Invitrogen, C1157) was used.

### Fluorescence in situ hybridization (FISH) and confocal microscopy

HRT18 cells were seeded on glass slides in a 24-well plate at 50% confluency and grown for 4 days until full confluency. The cells were then transferred to the anaerobic chamber, washed twice with anaerobic DPBS, and infected with bacteria in DMEM medium without FCS at a MOI of 10 and 50 for 20 h at 37 °C in anaerobic conditions. Cells were washed 3 times with cold Dulbecco’s Phosphate Buffered Saline (DPBS, Sigma, D8537) with Mg^2+^ and Ca^2+^ (Sigma-Aldrich, D8662) and fixed with 4% cold paraformaldehyde in PBS (PFA, VWR, J19943) for 30 min at room temperature (RT) and stopped by incubation with 50 mM NH_4_Cl in PBS for 10 min, all at RT. Attached bacteria was permeabilized for 1 h at 37 °C by placing the glass slides upside down in 30 µL of permeabilization buffer containing 25 mM Tris pH 7.5 (Life technologies, 15504020), 10 mM EDTA (Carl Roth, 1410.2), 585 mM sucrose (Merck Millipore, 84100), 5 mM CaCl_2_ (Sigma, 746495), 0.3 mg/mL sodium deoxycholate (Merck Millipore, VL683704), and 3 mg/mL lysozyme (Sigma, L6876). Glass slides were stained by placing them upside down in a 30 µL droplet hybridization buffer containing 0.9 M NaCl (Merck, 1064041000), 20 mM Tris pH 7.5, 0.1% SDS (Invitrogen, 15553027), and 20% formamide with 1000 nM EUB338-AF488 probe (with sequence 5’- GCTGCCTCCCGTAGGAGT-3/Alexa488N’ and T_M_ 59.4 °C). After 2 h incubation at 50 °C in a humidity chamber, the slides were washed by immersing in 1 mL hybridization buffer for 15 min at room temperature (RT) and twice with 1 mL DPBS + 0.2% BSA (Sigma, A7030) for 5 min. Then, slides were stained with DAPI at 1:1000 (Invitrogen, D21490) for 10 min and washed 3 times with DPBS for 15 min in total. The slides were shortly immersed in MilliQ water, and embedded in Prolong diamond mounting solution (Invitrogen, P36990). Imaging on a Leica SPE-II confocal microscope in combination with Leica LAS AF software. Image analysis was performed using Fiji/ImageJ. At least three biological replicates were performed.

### Induction of soluble mucus layer in Caco-2 cells and bacterial infection

24-well plates Transwells with a polyester (PET) membrane of 0.4 µM pore size (Sarstedt, 83.3932.041) were coated with collagen Type I (Corning, 354249) for 2 h at 37 °C. Subsequently, wells were washed with DPBS, let to air dry for 20 min and Caco-2 cells were seeded at 100% confluency. After three days incubation, the liquid from the upper compartment was taken off to create an air–liquid interface culture condition. The bottom compartment was refreshed every day with new media and 300 ng/mL of the Vasoactive Intestinal Peptide, VIP (Anaspec, AS-22872). After two weeks, cells were ready to be infected. Overnight bacteria were washed with PBS, set to OD = 1, and pelleted by centrifugation. The bacterial pellet was taken up in 60 µM pre-warmed CFSE and incubated for 45 min at 37 °C in the dark. Stained bacteria were washed three times by centrifugation with DMEM and subsequently used to infect the Caco-2 cells at MOI 50 for 4 h in anaerobic conditions.

### Immunofluorescence and confocal microscopy

For immunofluorescence, HRT18 cells were grown on coverslips in 24-well plates for 4 days, after which cells were infected with bacteria in DMEM medium without FCS at a MOI of 10 for 20 h at 37 °C in anaerobic conditions. StcE (in house) at 5 µg/mL for 20 h was used as a control for mucin *O*-glycan removal. Monolayers were washed twice with DPBS and fixed with 4% cold PFA for 30 min at RT. The fixation was stopped by incubation with 50 mM NH_4_Cl in PBS for 10 min at RT. Cells were washed twice with DPBS and permeabilized in binding buffer containing saponin (Sigma, S4521), BSA (Sigma, A7030), and DPBS for 30 min at RT. Subsequently, cells were incubated with primary antibodies (MUC13-CT at 1:100, ZO-1 at 1: 50 and occludin at 1:50) in binding buffer for 1 h at RT. For staining of the MUC13 extracellular domain, cells were incubated with MUC13-ED (undiluted) in 0.2% BSA in DPBS for 1 h at RT without prior permeabilization. Coverslips were washed 3 times with binding buffer followed by incubation with secondary antibodies (1:200) and DAPI (1:1000) for 1 h at RT. Coverslips were washed 3 times with DPBS, once with MilliQ-purified water (to remove salts that could interfere with microscopy visualization), and embedded in Prolong diamond mounting solution (Invitrogen, P36990). Images were collected on a Leica SPE-II confocal microscope in combination with Leica LAS AF software unless stated otherwise. Three biological replicates were performed for all immunofluorescence experiments.

To stain the mucus layer in the Caco-2 model, Caco-2 cells were seeded on collagen-coated 24-well Transwell plates as described before. After infection with CFSE-labelled bacteria, Caco-2 cells were fixed with 150 µL Carnoy’s solution composed of 60% ethanol (VWR, 1.009.831.000), 30% chloroform (VWR, 1.02442.1000) and 10% acetic acid (Enmsure, 1000621000) at the apical compartment for 2 h at RT. Then, the cells were washed with 100% ethanol and 150 µL 80% ethanol was added apically for 15 min and stored in PBS until further use. The mucus layer was stained with Jacalin (1:100) in blocking butter (2% BSA in PBS) for 1 h at RT. Subsequently, membranes were washed three times with blocking buffer for 5 min each and stained with DAPI (1:500) in blocking buffer for 30 min at RT. After three washes, membranes were cut, mounted onto microscope slides with the cells looking up, and embedded in Prolong diamond solution. Slides were imaged on the Nikon A1R/STORM and analysed using the NIS-Element Imaging software.

### Adhesion assay and quantification by CFU counting

Overnight bacterial cultures were washed with Dulbecco’s Phosphate Buffered Saline (DPBS, Sigma, D8537) and resuspended in Bifidobacterial medium. HRT18 cells were seeded in a 6-well plate and grown until full confluency. The cells were then transferred to the anaerobic chamber, washed twice with anaerobic DPBS to remove remaining oxygen, and infected with bacteria at a MOI of 10 and 50. After 2 h incubation at 37 °C, cells were washed 2 times with DPBS, incubated with 500 µL 0.25% trypsin (ThermoFisher, 25200-072) for 10 min at room temperature, and stopped with 500 µL of Dulbecco’s modified Eagle’s medium (DMEM) + glutamax (Life Technologies, 31966047) containing 10% fetal calf serum (FCS) (Sigma, F7524). The cell suspension was used to make tenfold serial dilutions. 100 μL aliquots from undiluted to the 10^–5^ dilution were plated and colonies were grown at 37 °C for 1 or 2 days under anaerobic conditions. For all bacterial strains, visual colonies from dilutions 10^–3^ or 10^–4^ were counted to determine CFU/mL.

### Immunoblotting

HRT18 cells were seeded on 6-well plates at 30% confluency and grown for 4 days until full confluency. The cells were then transferred to the anaerobic chamber, washed twice with anaerobic DPBS, and infected with bacteria in DMEM medium without FCS for up to 20 h at 37 °C in anaerobic conditions. Cell pellets were taken up in 1% SDS in PBS and lysed by mechanical lysis with a metal rack. Protein concentration was determined using a Pierce^™^ BCA Protein Assay kit and equal amounts of protein were prepared in Laemmli sample buffer and boiled for 5 min at 96 °C. For immunoblotting of MUC13, protein lysates were loaded onto a 10% SDS-PAGE gel and transferred to a 0.2 µm PVDF membrane using the Trans-Blot Turbo Transfer System (Biorad) for 10 min at 25 V and 1,3 amperes (High MW protocol). The membranes were blocked with 5% skimmed milk powder in PBS-Tween for 1 h at RT. Subsequently, the membranes were incubated with MUC13 (Abcam, 1:1000) antibody in PBS-Tween containing 1% skimmed milk powder o/n at 4 °C. The next day, the membranes were washed 4 times with PBS-Tween (10 min each) and incubated with secondary antibody diluted 1:5000 in PBS-Tween containing 1% skimmed milk powder for 1 h at RT. For immunoblotting of other proteins, protein lysates were loaded onto 8–12% SDS-PAGE gels and transferred to PVDF membranes using the Trans-Blot Turbo Transfer System (Biorad) for 7 min at 25 V and 1,3 amperes (High MW protocol). Blocking was done in 5% BSA-TSMT (20 mM Tris, 150 mM NaCl, 1 mM CaCl_2_, 2 mM MgCl_2_ adjusted to pH 7 with HCl and 0.1% Tween 20) for 1 h at RT. Antibodies were diluted in 1% BSA-TSMT and incubated o/n at 4 °C. Membranes were probed with claudin-1, -3, and -4 (1:500) antibodies and β-actin (1:2000) or GAPDH antibodies (1:1000). For visualization, blots were incubated with Clarity Western ECL or Femto ECL solutions (Biorad) and imaged in the Amersham ImageQuant 800 system (Cytiva).

For immunoblotting on HRT18 and Caco-2 cells grown on Transwells, cells were washed one time with 150 µL DPBS after bacterial infection. A volume of 100 µL DPBS was added, and cells were detached from the membrane by using a pipette tip. Subsequently, cells were lysed with 1% SDS and blots were stained with antibodies as previously described. All immunoblots used for analysis are available in Figure S1–S7.

### Expression of MUC13-GFP in HRT18 cells and MUC13 pull-down

HRT18-∆MUC13 cells expressing the C-ter domain of MUC13 tagged with GFP (HRT18-∆MUC13 + pMUC13)^8^ were seeded in 6 well plates and grown until full confluency. MUC13-eGFP expression was induced by addition of 20 ng/ml of doxycycline (Sigma, D3072) every other day for 6 days and observed under a fluorescent microscope for GFP signal. The cells were then transferred to the anaerobic chamber, washed twice with anaerobic DPBS, and infected with bacteria in DMEM medium without FCS for up to 2–5 h (depending on the experiment) at 37 °C in anaerobic conditions. To inhibit sialidases, we added 1.5 or 5.5 mM of N-acetyl-2,3-dehydro-2-deoxyneuraminic acid (DANA) (Sigma-Aldrich D9050) depending on the experiment. To block the action of proteases, 1 × Halt protease and phosphatase inhibitor cocktail (Thermo Scientific, 78441) was added. Cells were treated with 200 U/mL of α2,3,6,8,9 neuraminidase A (NEB Bioke, P0722L) and 0.6 U of α1,2,3,4,6-L-fucosidase (Megazyme, E-FUCHS) to evaluate the effect on MUC13 modification. Cells were washed with ice-cold PBS, harvested using a cell scraper, and lysed in lysing buffer containing 1% NP40, 25 mM Tris HCl pH 7.5, 150 mM NaCl, and 0.5 mM EDTA (Carl Roth, 1410.2). After overnight incubation at 4 °C, tubes were centrifuged at 17,000 xg for 10 min, protein concentration was measured, and equal amounts were added to 25 µL pre-equilibrated anti-GFP agarose magnetics beads (NanoTag, N0310) and incubated for 1 h rotating at 4 °C. Beads were washed 3 times with 1 mL of cold washing buffer (0.1% NP40, 25 mM Tris HCl pH 7.5, 150 mM NaCl, and 0.5 mM EDTA) using a magnetic holder. Samples were transferred to new Eppendorf’s before the last wash. To elute the antibody from the beads, samples were boiled in Laemmli Buffer, and analyzed by western blotting.

### Transepithelial electrical resistance (TEER) measurements

HRT18 cells were seeded in 12-well Transwell plates with 12 mm inserts and 0.4 µM membrane pore size (Costar, 3401) at 50% confluency. Wells without cells were taken along as negative control. Transepithelial electrical resistance was determined with a Millicell ERS-2 Voltohmmeter (Millipore). TEER measurements were taken every 2–3 days for 2 weeks to verify tight junction formation over time. Media in the basolateral compartment was replaced with fresh DMEM without FCS. Bacteria in DMEM were added at MOI 100 at the apical side and incubated for 2 h. Subsequently, 100 ng/mL of each IL-1β and TNF-α were added to the apical compartment and TEER was measured at different time points up to 28 h. After the last TEER measurement, several wells were fixed with 4% PFA and stained with occludin (1:50) and ZO-1 (1:100) to visualize the tight junctions. Cells were harvested from the remaining wells to determine claudin-1, -3, -4 (1:500), and MUC13 (1:1000) levels by western blotting. All measurements were performed on at least two individual wells and from three independent biological replicates. TEER (Ohm*cm^2^) values were calculated by subtracting the average negative control value from the measurement and multiplying it by the well surface (1.12 cm^2^).

### CAZyme analysis

The predicted protein sequence from DSMZ 20456 was obtained from the Uniprot database (uniprot.org/taxonomy/500634). Predicted bacterial protein sequences were used to analyze the presence of carbohydrate-active enzymes using the CAZy database and dbCAN3 meta server (https://bcb.unl.edu/dbCAN2/blast.php). CAZymes identified with at least two out of three tools (HMMER: dbCAN, DIAMOND: CAZy, and HMMER: dbCAN_sub) were considered for further analysis. Signal IP6 server (https://services.healthtech.dtu.dk/services/SignalP-6.0/) was used to predict the presence of signal peptides in the identified proteins.

### Enzymatic activity assays

Bacteria were grown overnight or until they reached stationary phase and washed three times in DPBS by centrifugation at 5,000 × g for 10 min. Pellets were diluted to OD600 = 1 in DPBS. Fucosidase activity was measured by adding 100 µM 4-Methylumbelliferyl α-L-fucopyranoside substrate (Sigma-Aldrich, M8527) to 50 µL of samples and incubated for 1 h at 37 °C in the dark. Fluorescence was measured at 340 nm (excitation) and 490 nm (emission), and a gain of 1124 (FLUOstar Omega). As a positive control for fucosidase activity, 0.6 Units of the commercial α1,2,3,4,6-l-fucosidase (Megazyme, E-FUCHS) was used. As a negative control, samples were pre-incubated with 100 µM of the fucosidase inhibitor L-fuconojirimycin (FNJ) (Carbosynth, CAS 99212-30-3) for 30 min at 37 °C. Sialidase activity was measured by adding 100 µM 4-Methylumbelliferyl N-acetyl-a-D-neuraminic acid sodium salt (Sigma, M8639) as a substrate. As a negative control, samples were pre-incubated with 1 mM of the sialidase inhibitor N-acetyl-2,3-dehydro-2-deoxyneuraminic acid (DANA) (Sigma-Aldrich D9050) for 30 min at 37 °C. After the addition of the substrate, the plates were incubated for 30 min at 37 °C in the dark. Fluorescence was measured at 340 nm (excitation) and 490 nm (emission) with a gain of 1124 (CLARIOstar Plus). For all assays, measured values for DPBS were subtracted from the sample measurements.

### Statistical analysis

Statistical analyses were performed using Graph Pad Prism 10 software. The Kolmogorov–Smirnov test was used to assess normality of the data. For immunoblot analyses in HRT18 cells (in Fig. 1E, G, Fig. 4D), the control sample was assigned a value of 1. Subsequent samples were quantified relative to the control and normalized against the reference protein GAPDH or actin, followed by a one-sample t-test. For immunoblots of Fig. 1B, C, we included an additional control sample for comparison to our non-treated condition, followed by a one-way ANOVA with Dunnett’s post hoc test. Western blots from Caco-2 cells, normalized to the reference protein actin, were evaluated using one-way ANOVA (analysis of variance) with Dunnett’s post hoc test to compare each bacterial sample to the uninfected control. Sialidase activity data were analyzed using an independent *t*-test. The effect of bacterial treatment on TEER was evaluated using two-way ANOVA with Dunnett’s post hoc test. All graphs depict the mean and standard error of the mean (SEM) of at least three independent experiments. A *p*-value of < 0.05 was considered significant. * *p* < 0.05; ** *p* < 0.01; *** *p* < *0.001*. **** *p* < 0.0001.

## Supplementary Information

Supplementary Information.

## Data Availability

All data are provided within the manuscript or supplementary information files.

## References

[1] Johansson, M. E. V. et al. Normalization of host intestinal mucus layers requires long-term microbial colonization. *Cell Host Microbe* **18**, 582–592 (2015). 26526499 10.1016/j.chom.2015.10.007 PMC4648652

[2] Johansson, M. E. V., Larsson, J. M. H. & Hansson, G. C. The two mucus layers of colon are organized by the MUC2 mucin, whereas the outer layer is a legislator of host-microbial interactions. *Proc. Natl. Acad. Sci. U. S. A.* **108**(Suppl 1), 4659–4665 (2011). 20615996 10.1073/pnas.1006451107 PMC3063600

[3] Javitt, G. et al. Assembly mechanism of mucin and von Willebrand factor polymers. *Cell* **183**, 717-729.e16 (2020). 33031746 10.1016/j.cell.2020.09.021 PMC7599080

[4] van Putten, J. P. M. & Strijbis, K. Transmembrane mucins: Signaling receptors at the intersection of inflammation and cancer. *J. Innate Immun.* **9**, 281–299 (2017). 28052300 10.1159/000453594 PMC5516414

[5] Akita, K. et al. CA125/MUC16 interacts with Src family kinases, and over-expression of its C-terminal fragment in human epithelial cancer cells reduces cell-cell adhesion. *Eur. J. Cell Biol.* **92**, 257–263 (2013). 24246580 10.1016/j.ejcb.2013.10.005

[6] Gao, C., Xiao, G. & Hu, J. Regulation of Wnt/β-catenin signaling by posttranslational modifications. *Cell Biosci.* **4**, 13 (2014). 24594309 10.1186/2045-3701-4-13 PMC3977945

[7] Huang, L. et al. MUC1 oncoprotein blocks glycogen synthase kinase 3beta-mediated phosphorylation and degradation of beta-catenin. *Cancer Res.* **65**, 10413–10422 (2005). 16288032 10.1158/0008-5472.CAN-05-2474

[8] Segui-Perez, C. et al. MUC13 negatively regulates tight junction proteins and intestinal epithelial barrier integrity via protein kinase C. *J. Cell Sci.* **137**, jcs261468 (2024). 38345099 10.1242/jcs.261468 PMC10984281

[9] Breugelmans, T. et al. In-depth study of transmembrane mucins in association with intestinal barrier dysfunction during the course of T cell transfer and DSS-induced colitis. *J. Crohns Colitis* **14**, 974–994 (2020). 32003421 10.1093/ecco-jcc/jjaa015

[10] Turroni, F. et al. Genome analysis of Bifidobacterium bifidum PRL2010 reveals metabolic pathways for host-derived glycan foraging. *Proc. Natl. Acad. Sci. U. S. A.* **107**, 19514–19519 (2010). 20974960 10.1073/pnas.1011100107 PMC2984195

[11] Turroni, F. et al. Diversity of bifidobacteria within the infant gut microbiota. *PLoS One* **7**, e36957 (2012). 22606315 10.1371/journal.pone.0036957 PMC3350489

[12] Golubkova, A. & Hunter, C. J. Development of the neonatal intestinal barrier, microbiome, and susceptibility to NEC. *Microorganisms* **11**, 1247 (2023). 37317221 10.3390/microorganisms11051247 PMC10221463

[13] Borewicz, K. et al. Correlating infant fecal microbiota composition and human milk oligosaccharide consumption by microbiota of 1-month-old breastfed infants. *Mol. Nutr. Food Res.* **63**, e1801214 (2019). 31017343 10.1002/mnfr.201801214 PMC6618098

[14] Ward, R. E., Niñonuevo, M., Mills, D. A., Lebrilla, C. B. & German, J. B. In vitro fermentability of human milk oligosaccharides by several strains of bifidobacteria. *Mol. Nutr. Food Res.* **51**, 1398–1405 (2007). 17966141 10.1002/mnfr.200700150

[15] Milani, C. et al. The first microbial colonizers of the human gut: Composition, activities, and health implications of the infant gut microbiota. *Microbiol. Mol. Biol. Rev.* **81**, e00036-e117 (2017). 29118049 10.1128/MMBR.00036-17 PMC5706746

[16] Yatsunenko, T. et al. Human gut microbiome viewed across age and geography. *Nature* **486**, 222–227 (2012). 22699611 10.1038/nature11053 PMC3376388

[17] Bunesova, V., Lacroix, C. & Schwab, C. Mucin cross-feeding of infant Bifidobacteria and Eubacterium hallii. *Microb. Ecol.* **75**, 228–238 (2018). 28721502 10.1007/s00248-017-1037-4

[18] Egan, M. et al. Cross-feeding by Bifidobacterium breve UCC2003 during co-cultivation with Bifidobacterium bifidum PRL2010 in a mucin-based medium. *BMC Microbiol.* **14**, 282 (2014). 25420416 10.1186/s12866-014-0282-7 PMC4252021

[19] Moens, F., Weckx, S. & De Vuyst, L. Bifidobacterial inulin-type fructan degradation capacity determines cross-feeding interactions between bifidobacteria and Faecalibacterium prausnitzii. *Int. J. Food Microbiol.* **231**, 76–85 (2016). 27233082 10.1016/j.ijfoodmicro.2016.05.015

[20] López, P. et al. Interaction of Bifidobacterium bifidum LMG13195 with HT29 cells influences regulatory-T-cell-associated chemokine receptor expression. *Appl. Environ. Microbiol.* **78**, 2850–2857 (2012). 22344636 10.1128/AEM.07581-11 PMC3318848

[21] Waller, P. A. et al. Dose-response effect of Bifidobacterium lactis HN019 on whole gut transit time and functional gastrointestinal symptoms in adults. *Scand. J. Gastroenterol.* **46**, 1057–1064 (2011). 21663486 10.3109/00365521.2011.584895 PMC3171707

[22] Corrêa, N. B. O., Péret Filho, L. A., Penna, F. J., Lima, F. M. L. S. & Nicoli, J. R. A randomized formula controlled trial of Bifidobacterium lactis and Streptococcus thermophilus for prevention of antibiotic-associated diarrhea in infants. *J. Clin. Gastroenterol.* **39**, 385–389 (2005). 15815206 10.1097/01.mcg.0000159217.47419.5b

[23] Selinger, C. P. et al. Probiotic VSL#3 prevents antibiotic-associated diarrhoea in a double-blind, randomized, placebo-controlled clinical trial. *J. Hosp. Infect.* **84**, 159–165 (2013). 23618760 10.1016/j.jhin.2013.02.019

[24] Holmén Larsson, J. M., Thomsson, K. A., Rodríguez-Piñeiro, A. M., Karlsson, H. & Hansson, G. C. Studies of mucus in mouse stomach, small intestine, and colon. III. Gastrointestinal Muc5ac and Muc2 mucin O-glycan patterns reveal a regiospecific distribution. *Am. J. Physiol. Gastrointest. Liver Physiol.* **305**, G357–G363 (2013). 23832516 10.1152/ajpgi.00048.2013 PMC3761246

[25] Robbe, C., Capon, C., Flahaut, C. & Michalski, J.-C. Microscale analysis of mucin-type O-glycans by a coordinated fluorophore-assisted carbohydrate electrophoresis and mass spectrometry approach. *Electrophoresis* **24**, 611–621 (2003). 12601728 10.1002/elps.200390071

[26] Van Klinken, B. J., Dekker, J., Büller, H. A. & Einerhand, A. W. Mucin gene structure and expression: protection vs. adhesion. *Am. J. Physiol.* **269**, G613-627 (1995). 7491952 10.1152/ajpgi.1995.269.5.G613

[27] Tailford, L. E., Crost, E. H., Kavanaugh, D. & Juge, N. Mucin glycan foraging in the human gut microbiome. *Front. Genet.*10.3389/fgene.2015.00081 (2015). 25852737 10.3389/fgene.2015.00081 PMC4365749

[28] Midtvedt, A. C., Carlstedt-Duke, B. & Midtvedt, T. Establishment of a mucin-degrading intestinal microflora during the first two years of human life. *J. Pediatr. Gastroenterol. Nutr.* **18**, 321–326 (1994). 8057215 10.1097/00005176-199404000-00012

[29] Glover, J. S., Ticer, T. D. & Engevik, M. A. Characterizing the mucin-degrading capacity of the human gut microbiota. *Sci. Rep.* **12**, 8456 (2022). 35589783 10.1038/s41598-022-11819-z PMC9120202

[30] Ruas-Madiedo, P., Gueimonde, M., Fernández-García, M., Reyes-Gavilán, C. G. & Margolles, A. Mucin degradation by Bifidobacterium strains isolated from the human intestinal microbiota. *Appl. Environ. Microbiol.* **74**, 1936–1940 (2008). 18223105 10.1128/AEM.02509-07 PMC2268317

[31] Engevik, M. A. et al. Bifidobacterium dentium fortifies the intestinal mucus layer via autophagy and calcium signaling pathways. *mBio* **10**, e01087-e1119 (2019). 31213556 10.1128/mBio.01087-19 PMC6581858

[32] Javed, N. H., Alsahly, M. B. & Khubchandani, J. Oral feeding of probiotic Bifidobacterium infantis: Colonic morphological changes in rat model of TNBS-induced colitis. *Scientifica* **2016**, 9572596 (2016). 27127686 10.1155/2016/9572596 PMC4834163

[33] Schroeder, B. O. et al. Bifidobacteria or fiber protect against diet-induced microbiota-mediated colonic mucus deterioration. *Cell Host Microbe* **23**, 27-40.e7 (2018). 29276171 10.1016/j.chom.2017.11.004 PMC5764785

[34] Leitch, E. C. M., Walker, A. W., Duncan, S. H., Holtrop, G. & Flint, H. J. Selective colonization of insoluble substrates by human faecal bacteria. *Environ. Microbiol.* **9**, 667–679 (2007). 17298367 10.1111/j.1462-2920.2006.01186.x

[35] Raimondi, S., Musmeci, E., Candeliere, F., Amaretti, A. & Rossi, M. Identification of mucin degraders of the human gut microbiota. *Sci. Rep.* **11**, 11094 (2021). 34045537 10.1038/s41598-021-90553-4 PMC8159939

[36] Milatz, S. et al. Claudin-3 acts as a sealing component of the tight junction for ions of either charge and uncharged solutes. *Biochim. Biophys. Acta* **1798**, 2048–2057 (2010). 20655293 10.1016/j.bbamem.2010.07.014

[37] Katayama, T. et al. Molecular cloning and characterization of Bifidobacterium bifidum 1,2-alpha-L-fucosidase (AfcA), a novel inverting glycosidase (glycoside hydrolase family 95). *J. Bacteriol.* **186**, 4885–4893 (2004). 15262925 10.1128/JB.186.15.4885-4893.2004 PMC451662

[38] Zhang, H. et al. dbCAN2: a meta server for automated carbohydrate-active enzyme annotation. *Nucleic Acids Res.* **46**, W95–W101 (2018). 29771380 10.1093/nar/gky418 PMC6031026

[39] Bai, X., Brown, J. R., Varki, A. & Esko, J. D. Enhanced 3-O-sulfation of galactose in Asn-linked glycans and Maackia amurenesis lectin binding in a new Chinese hamster ovary cell line. *Glycobiology* **11**, 621–632 (2001). 11479273 10.1093/glycob/11.8.621

[40] Geisler, C. & Jarvis, D. L. Letter to the glyco-forum: Effective glycoanalysis with Maackia amurensis lectins requires a clear understanding of their binding specificities. *Glycobiology* **21**, 988 (2011). 21863598 10.1093/glycob/cwr080 PMC3130539

[41] Floor, E. et al. Development of a Caco-2-based intestinal mucosal model to study intestinal barrier properties and bacteria–mucus interactions. *Gut Microbes*10.1080/19490976.2024.2434685 (2025). 39714032 10.1080/19490976.2024.2434685 PMC11702969

[42] Hemert, S. V. & Ormel, G. Influence of the multispecies probiotic Ecologic® BARRIER on parameters of intestinal barrier function. *Food Nutr. Sci.* **5**, 1739–1745 (2014).

[43] Karczewski, J. et al. Regulation of human epithelial tight junction proteins by Lactobacillus plantarum in vivo and protective effects on the epithelial barrier. *Am. J. Physiol. Gastrointest. Liver Physiol.* **298**, G851-859 (2010). 20224007 10.1152/ajpgi.00327.2009

[44] Al-Sadi, R. et al. Bifidobacterium bifidum enhances the intestinal epithelial tight junction barrier and protects against intestinal inflammation by targeting the toll-like receptor-2 pathway in an NF-κB-independent manner. *Int. J. Mol. Sci.* **22**, 8070 (2021). 34360835 10.3390/ijms22158070 PMC8347470

[45] Chichlowski, M., De Lartigue, G., German, J. B., Raybould, H. E. & Mills, D. A. Bifidobacteria isolated from infants and cultured on human milk oligosaccharides affect intestinal epithelial function. *J. Pediatr. Gastroenterol. Nutr.* **55**, 321–327 (2012). 22383026 10.1097/MPG.0b013e31824fb899 PMC3381975

[46] Hsieh, C.-Y. et al. Strengthening of the intestinal epithelial tight junction by Bifidobacterium bifidum. *Physiol. Rep.* **3**, e12327 (2015). 25780093 10.14814/phy2.12327 PMC4393161

[47] Cassard, L. et al. Individual strains of Lactobacillus paracasei differentially inhibit human basophil and mouse mast cell activation. *Immunity Inflamm. Dis.* **4**, 289–299 (2016). 10.1002/iid3.113 PMC5004284 27621812

[48] He, F. et al. Adhesion of Bifidobacterium Spp. to human intestinal mucus. *Microbiol. Immunol.* **45**, 259–262 (2001). 11345536 10.1111/j.1348-0421.2001.tb02615.x

[49] Wang, J. et al. Probiotic Lactobacillus plantarum promotes intestinal barrier function by strengthening the epithelium and modulating gut microbiota. *Front. Microbiol.* **9**, 1953 (2018). 30197632 10.3389/fmicb.2018.01953 PMC6117384

[50] Sakanaka, M. et al. Varied pathways of infant gut-associated Bifidobacterium to assimilate human milk oligosaccharides: Prevalence of the gene set and its correlation with Bifidobacteria-rich microbiota formation. *Nutrients* **12**, 71 (2019). 31888048 10.3390/nu12010071 PMC7019425

[51] Asakuma, S. et al. Physiology of consumption of human milk oligosaccharides by infant gut-associated bifidobacteria. *J. Biol. Chem.* **286**, 34583–34592 (2011). 21832085 10.1074/jbc.M111.248138 PMC3186357

[52] Gotoh, A. et al. Sharing of human milk oligosaccharides degradants within bifidobacterial communities in faecal cultures supplemented with Bifidobacterium bifidum. *Sci. Rep.* **8**, 13958 (2018). 30228375 10.1038/s41598-018-32080-3 PMC6143587

[53] Paone, P. & Cani, P. D. Mucus barrier, mucins and gut microbiota: the expected slimy partners?. *Gut* **69**, 2232–2243 (2020). 32917747 10.1136/gutjnl-2020-322260 PMC7677487

[54] Ghotaslou, R. et al. The metabolic, protective, and immune functions of Akkermansia muciniphila. *Microbiol. Res.* **266**, 127245 (2023). 36347103 10.1016/j.micres.2022.127245

[55] Hagi, T. & Belzer, C. The interaction of Akkermansia muciniphila with host-derived substances, bacteria and diets. *Appl. Microbiol. Biotechnol.* **105**, 4833–4841 (2021). 34125276 10.1007/s00253-021-11362-3 PMC8236039

[56] Luo, Y. et al. Akkermansia muciniphila prevents cold-related atrial fibrillation in rats by modulation of TMAO induced cardiac pyroptosis. *EBioMedicine* **82**, 104087 (2022). 35797768 10.1016/j.ebiom.2022.104087 PMC9270211

[57] Johansson, M. E. V. et al. Bacteria penetrate the normally impenetrable inner colon mucus layer in both murine colitis models and patients with ulcerative colitis. *Gut* **63**, 281–291 (2014). 23426893 10.1136/gutjnl-2012-303207 PMC3740207

[58] Singh, N. & Bernstein, C. N. Environmental risk factors for inflammatory bowel disease. *United Eur. Gastroenterol. J.* **10**, 1047–1053 (2022). 10.1002/ueg2.12319 PMC9752273 36262056

[59] Gomi, A. et al. Effect of Bifidobacterium bifidum BF-1 on gastric protection and mucin production in an acute gastric injury rat model. *J. Dairy Sci.* **96**, 832–837 (2013). 23200466 10.3168/jds.2012-5950

[60] Kawahara, T. et al. Oral administration of Bifidobacterium bifidum G9–1 alleviates rotavirus gastroenteritis through regulation of intestinal homeostasis by inducing mucosal protective factors. *PLoS ONE* **12**, e0173979 (2017). 28346473 10.1371/journal.pone.0173979 PMC5367788

[61] Martín, R. et al. The infant-derived Bifidobacterium bifidum strain CNCM I-4319 strengthens gut functionality. *Microorganisms* **8**, 1313 (2020). 32872165 10.3390/microorganisms8091313 PMC7565306

[62] Yoshihara, T. et al. The protective effect of Bifidobacterium bifidum G9–1 against mucus degradation by Akkermansia muciniphila following small intestine injury caused by a proton pump inhibitor and aspirin. *Gut Microbes* **11**, 1385–1404 (2020). 32515658 10.1080/19490976.2020.1758290 PMC7527075

[63] Abdulqadir, R., Engers, J. & Al-Sadi, R. Role of Bifidobacterium in modulating the intestinal epithelial tight junction barrier: Current knowledge and perspectives. *Curr. Dev. Nutr.* **7**, 102026 (2023). 38076401 10.1016/j.cdnut.2023.102026 PMC10700415

[64] Monteagudo-Mera, A., Rastall, R. A., Gibson, G. R., Charalampopoulos, D. & Chatzifragkou, A. Adhesion mechanisms mediated by probiotics and prebiotics and their potential impact on human health. *Appl. Microbiol. Biotechnol.* **103**, 6463–6472 (2019). 31267231 10.1007/s00253-019-09978-7 PMC6667406

[65] Taverniti, V. & Guglielmetti, S. The immunomodulatory properties of probiotic microorganisms beyond their viability (ghost probiotics: proposal of paraprobiotic concept). *Genes Nutr.* **6**, 261–274 (2011). 21499799 10.1007/s12263-011-0218-x PMC3145061

[66] Resta-Lenert, S. & Barrett, K. E. Live probiotics protect intestinal epithelial cells from the effects of infection with enteroinvasive Escherichia coli (EIEC). *Gut* **52**, 988–997 (2003). 12801956 10.1136/gut.52.7.988 PMC1773702

[67] Guglielmetti, S. et al. Implication of an outer surface lipoprotein in adhesion of Bifidobacterium bifidum to Caco-2 cells. *Appl. Environ. Microbiol.* **74**, 4695–4702 (2008). 18539800 10.1128/AEM.00124-08 PMC2519326

[68] Serafini, F. et al. Evaluation of adhesion properties and antibacterial activities of the infant gut commensal *Bifidobacterium bifidum* PRL2010. *Anaerobe* **21**, 9–17 (2013). 23523946 10.1016/j.anaerobe.2013.03.003

[69] Nishiyama, K. et al. Bifidobacterium bifidum extracellular sialidase enhances adhesion to the mucosal surface and supports carbohydrate assimilation. *mBio* **8**, e00928-e1017 (2017). 28974612 10.1128/mBio.00928-17 PMC5626965

[70] Katoh, T. et al. Enzymatic adaptation of Bifidobacterium bifidum to host glycans, viewed from glycoside hydrolyases and carbohydrate-binding modules. *Microorganisms* **8**, 481 (2020). 32231096 10.3390/microorganisms8040481 PMC7232152

