# Supplementary material for: Probiotic Bifidobacterium bifidum strains desialylate MUC13 and increase intestinal epithelial barrier function
Source: Sci Rep. 2025 Mar 13;15:8778. doi: 10.1038/s41598-025-92125-2 (PMC11906825; doi:10.1038/s41598-025-92125-2)
Supplement: Supplementary file 1 — Supplementary Information. [file 41598_2025_92125_MOESM1_ESM.docx]

**
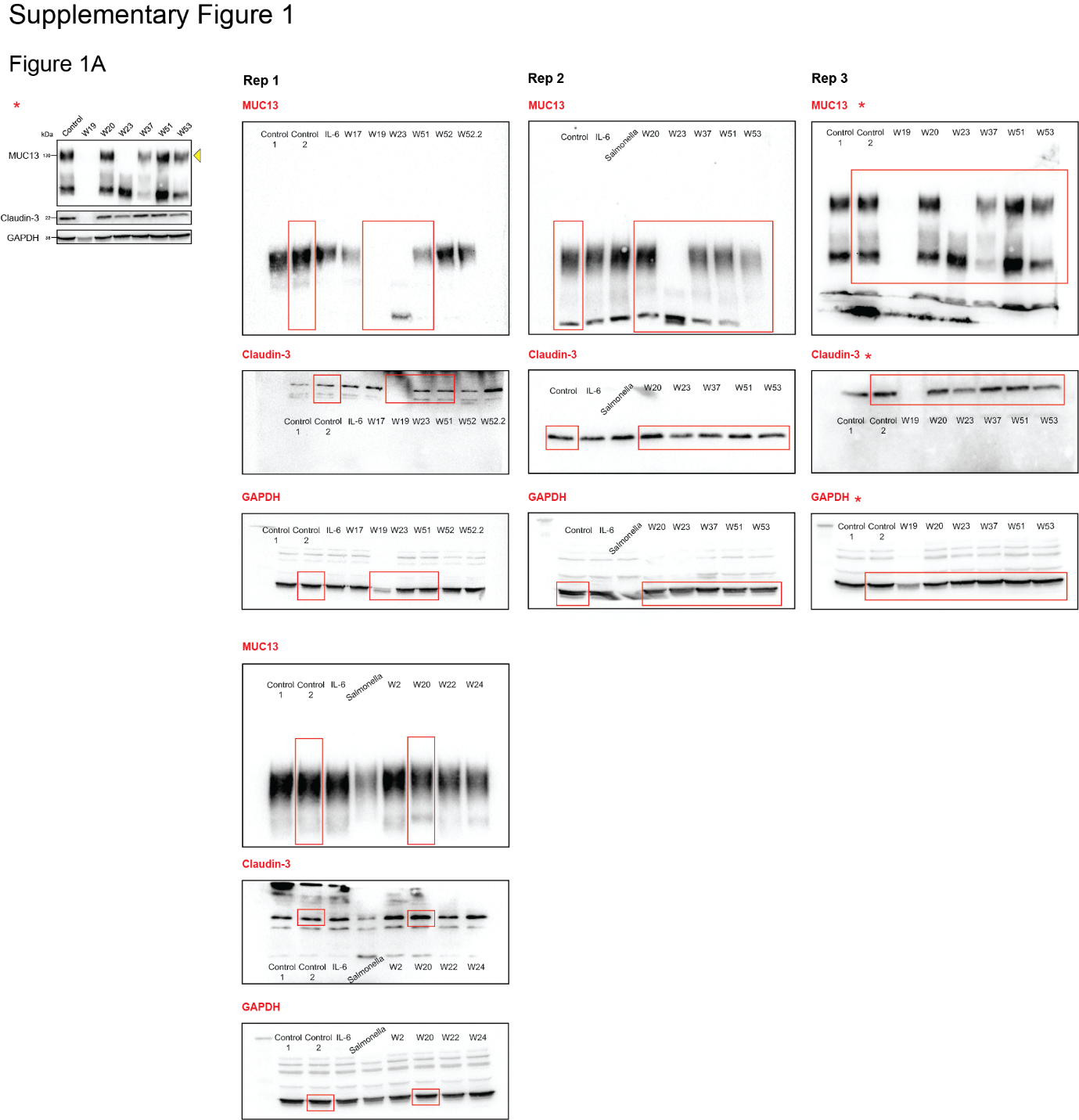
**

**Figure S1. Original immunoblot data used for Figure 1A.**

RAW immunoblot data of experimental replicates generated and analyzed for Figure 1A (left).


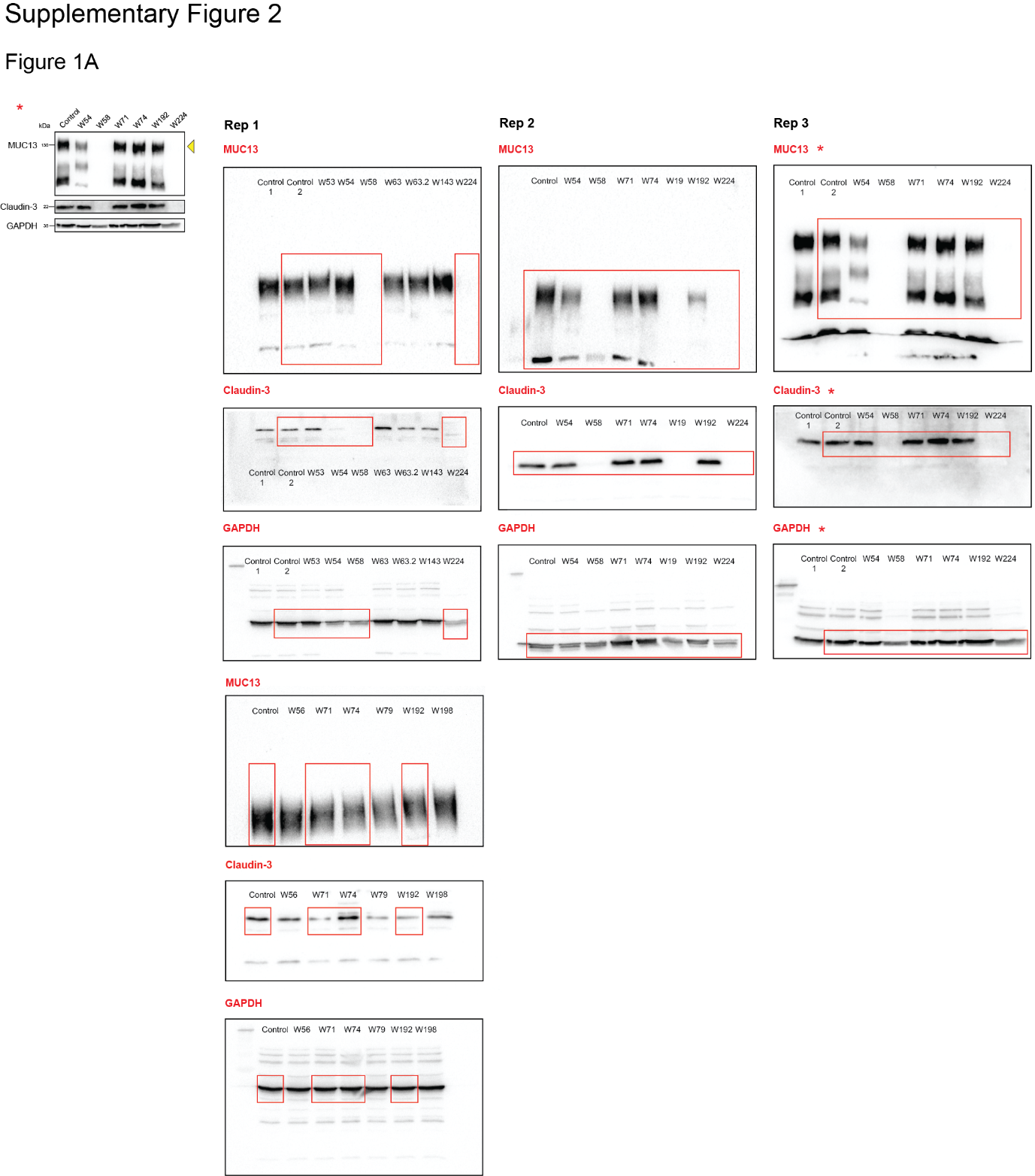


**Figure S2. Original immunoblot data Figure 1A.**

RAW immunoblot data generated and analyzed for Figure 1A (right).

**
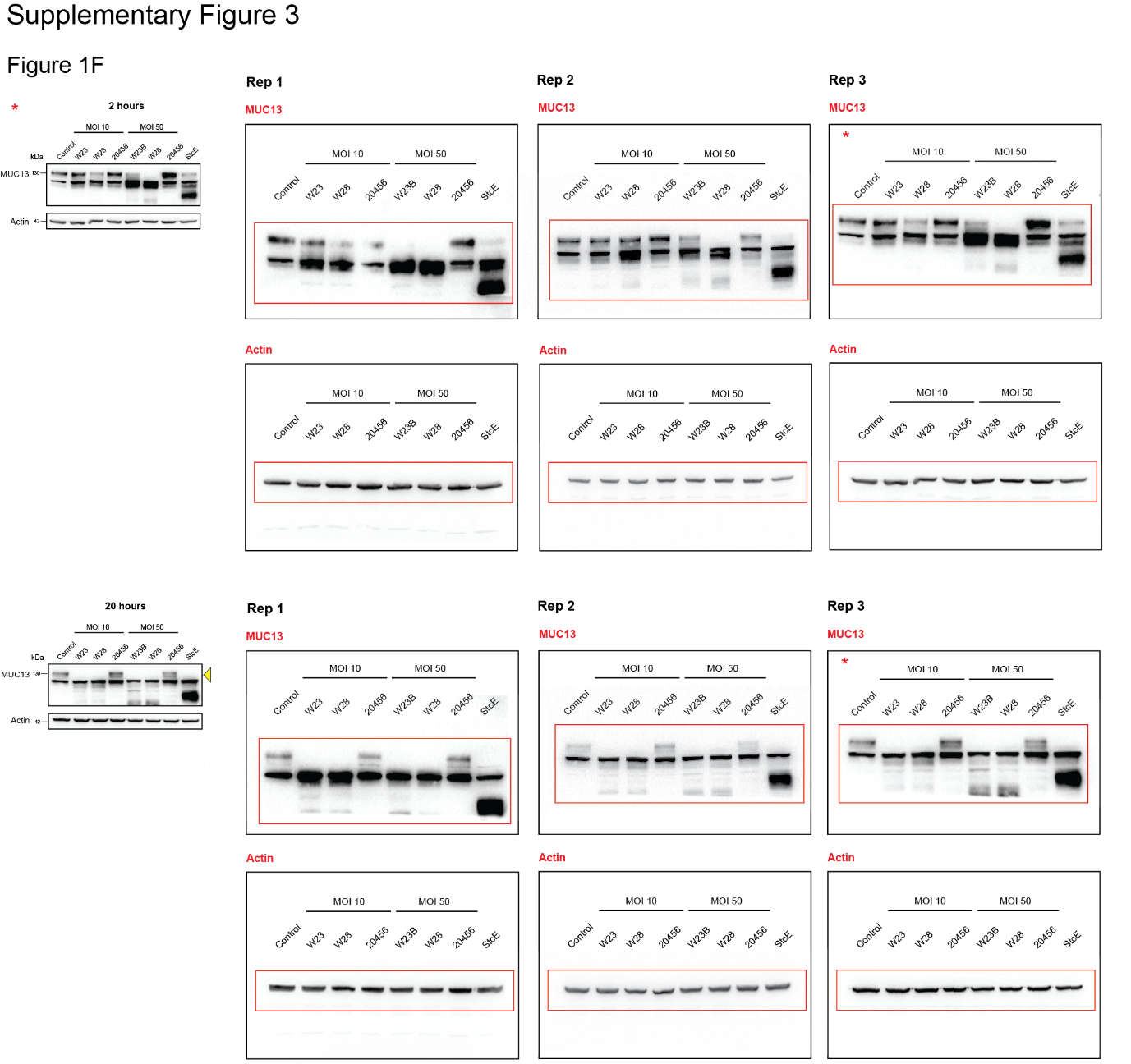
**

**Figure S3. Original immunoblot data Figure 1F.**

RAW immunoblot data of experimental replicates generated and analyzed for Figure 1F. Relevant areas are marked by red boxes.

**
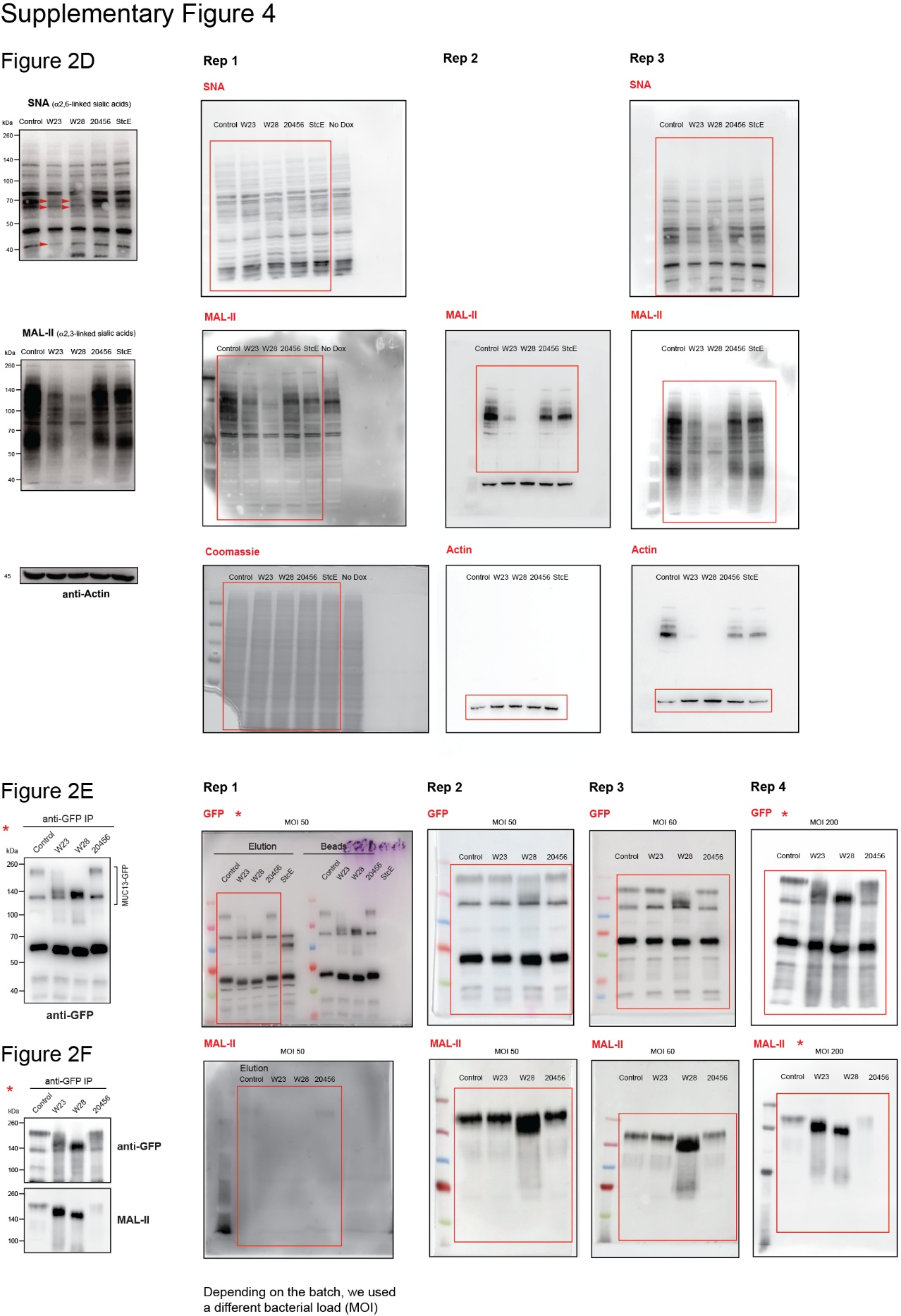
**

**Figure S4. Original immunoblot data Figures 2D-F.**

RAW immunoblot data of experimental replicates generated and analyzed for Figures 2D-F. Relevant areas are marked by red boxes.

**
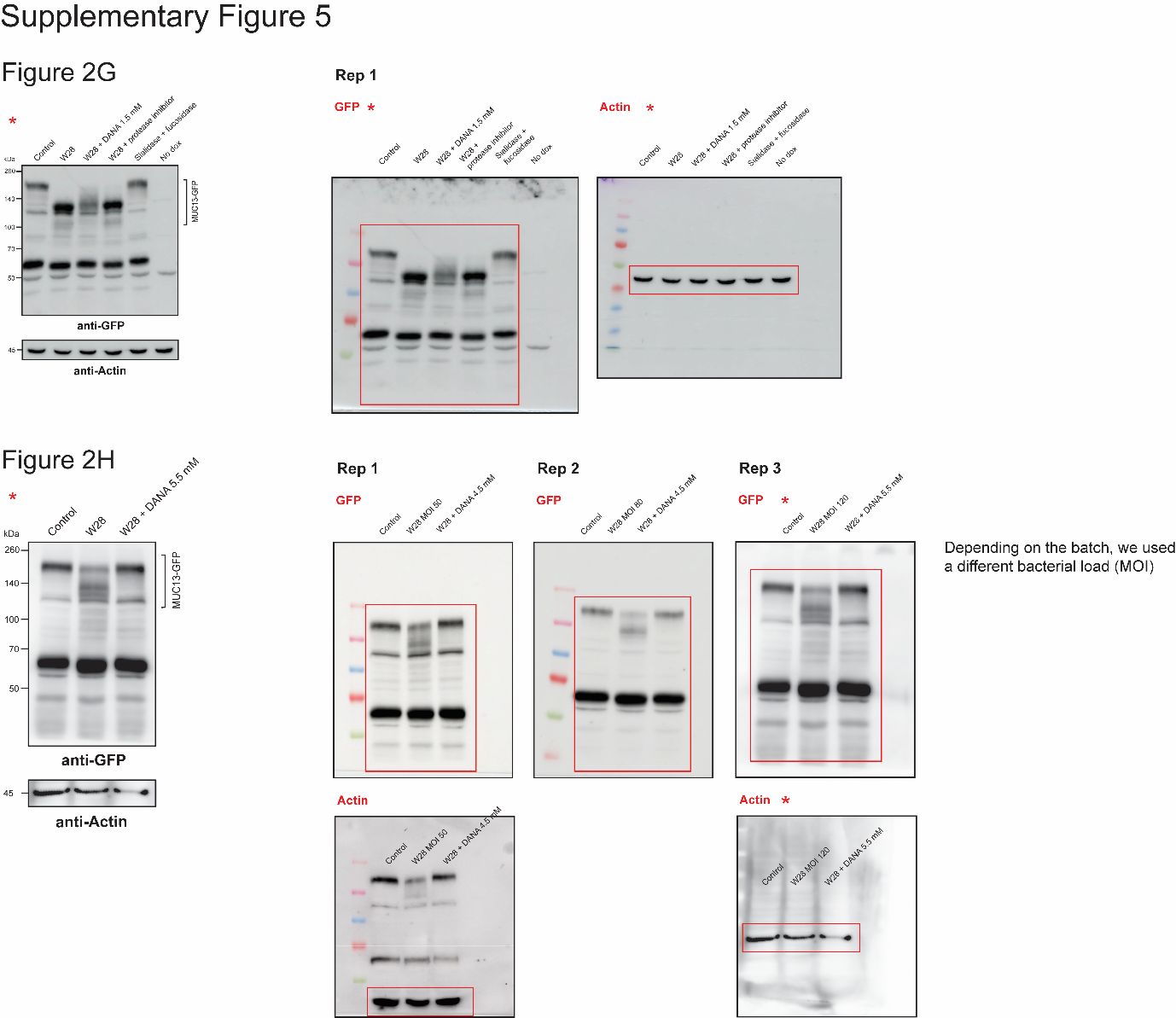
**

**Figure S5. Original immunoblot data Figures 2G-H.**

RAW immunoblot data of experimental replicates generated and analyzed for Figures 2G-H. Relevant areas are marked by red boxes.

**
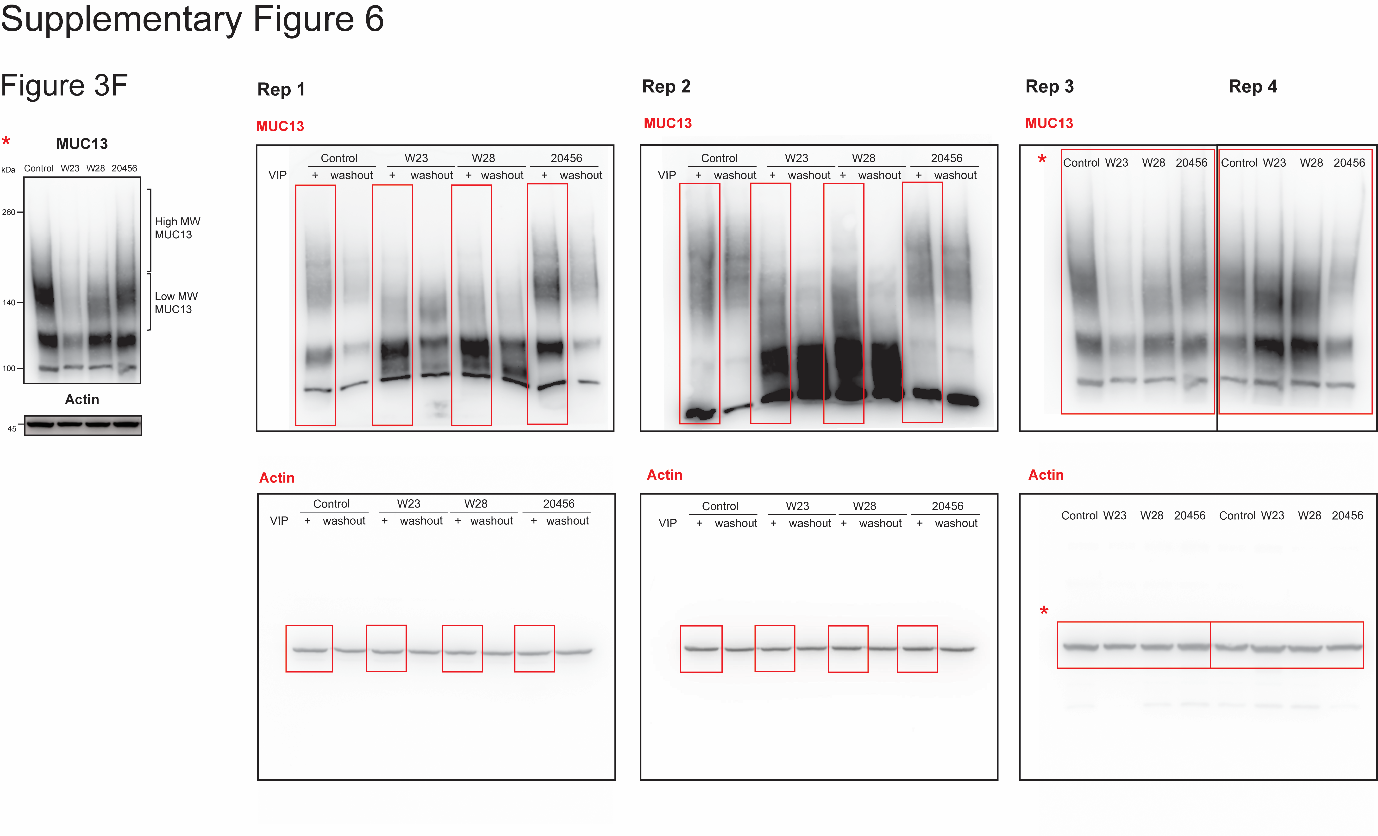
**

**Figure S6. Original immunoblot data Figure 3F.**

RAW immunoblot data of experimental replicates generated and analyzed for Figure 3F. Relevant areas are marked by red boxes.

**
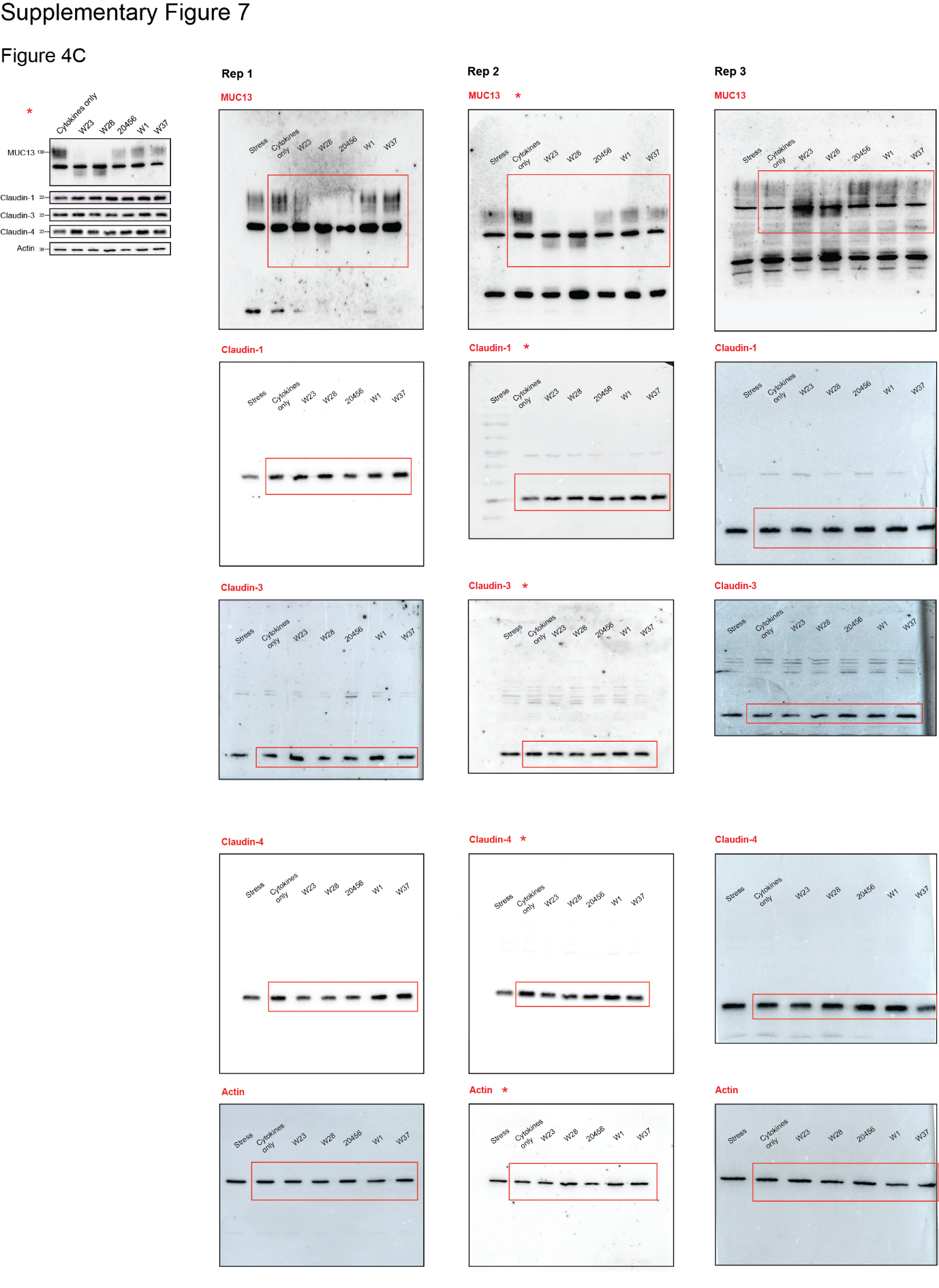
**

**Figure S7. Original immunoblot data Figure 4C.**

RAW immunoblot data of experimental replicates generated and analyzed for Figure 4C. Relevant areas are marked by red boxes.
